# Identification and validation of potential hypoxia-related genes associated with coronary artery disease

**DOI:** 10.3389/fphys.2023.1181510

**Published:** 2023-08-10

**Authors:** Yuqing Jin, Weiyan Ren, Jiayi Liu, Xuejiao Tang, Xinrui Shi, Dongchen Pan, Lianguo Hou, Lei Yang

**Affiliations:** ^1^ Department of Epidemiology, School of Public Health, Hebei Medical University, Shijiazhuang, China; ^2^ Biochemistry Research Laboratory, School of Basic Medicine, Hebei Medical University, Shijiazhuang, China

**Keywords:** hypoxia, coronary artery disease, metabolism, single-cell sequencing, immune cell infiltration

## Abstract

**Introduction:** Coronary artery disease (CAD) is one of the most life-threatening cardiovascular emergencies with high mortality and morbidity. Increasing evidence has demonstrated that the degree of hypoxia is closely associated with the development and survival outcomes of CAD patients. However, the role of hypoxia in CAD has not been elucidated.

**Methods:** Based on the GSE113079 microarray dataset and the hypoxia-associated gene collection, differential analysis, machine learning, and validation of the screened hub genes were carried out.

**Results:** In this study, 54 differentially expressed hypoxia-related genes (DE-HRGs), and then 4 hub signature genes (ADM, PPFIA4, FAM162A, and TPBG) were identified based on microarray datasets GSE113079 which including of 93 CAD patients and 48 healthy controls and hypoxia-related gene set. Then, 4 hub genes were also validated in other three CAD related microarray datasets. Through GO and KEGG pathway enrichment analyses, we found three upregulated hub genes (ADM, PPFIA4, TPBG) were strongly correlated with differentially expressed metabolic genes and all the 4 hub genes were mainly enriched in many immune-related biological processes and pathways in CAD. Additionally, 10 immune cell types were found significantly different between the CAD and control groups, especially CD8 T cells, which were apparently essential in cardiovascular disease by immune cell infiltration analysis. Furthermore, we compared the expression of 4 hub genes in 15 cell subtypes in CAD coronary lesions and found that ADM, FAM162A and TPBG were all expressed at higher levels in endothelial cells by single-cell sequencing analysis.

**Discussion:** The study identified four hypoxia genes associated with coronary heart disease. The findings provide more insights into the hypoxia landscape and, potentially, the therapeutic targets of CAD.

## 1 Introduction

Coronary artery disease (CAD) has severe morbidity and mortality globally (Chen et al., 2020). CAD comprises myocardial ischemia, anoxia, and even necrosis attributed to atherosclerosis of the coronary arteries, which results in myocardial injury and heart failure (HF) (Ties et al., 2022). Most importantly, the first manifestation of CAD may be either acute coronary syndrome (ACS) or sudden cardiac death (SCD) after rupture and thrombosis of an unstable non-obstructive atherosclerotic plaque, which was previously silent (Vahatalo et al., 2021). It is estimated that 540,000 people die of SCD every year in China and approximately 3 million people in the world every year (Refaat et al., 2015). Even with good equipments and well-trained professionals, the rescue success rate of SCD is still less than 5% (Zheng et al., 2022). Therefore, finding effective therapeutic targets is critical to the prevention and treatment of CAD.

Presently, the diagnoses of CAD, notably joint imaging techniques, are well-established. However, patients with an early stage of CAD are still difficult to diagnose because of the efficient and specific biomarkers deficiency (Wang et al., 2020; Su et al., 2021). Many studies believe that hypoxia is considered the initiating factor of fatal events in CAD patients, which involves complex pathways (Lucero Garcia Rojas et al., 2021; Ullah and Wu, 2021; Yu et al., 2022). Studies have found a direct relationship between chronic intermittent hypoxia and cardiovascular diseases such as atherosclerosis (AS) and sudden cardiac death (SCD) (AbdelMassih et al., 2021; Luo et al., 2022). Abnormalities in hypoxia-inducible factors (HIFs) have been found to be involved in a variety of cardiovascular injuries as the master regulators of the hypoxia response pathway (Ullah and Wu, 2021). The possible roles and specific molecular mechanisms remain to be fully elucidated. Therefore, to provide novel perspectives on prevention and treatment, more potential targets and hypoxia-related genes in CAD should be further explored.

The onset and progression of CAD may be significantly impacted by immune cell infiltration, according to a growing number of studies. By encouraging the recruitment of innate immune cells and interfering with the differentiation and function of adaptive immune cells, prior research has demonstrated that hypoxia can control immune cell infiltration in malignancies (Palazon et al., 2014). However, the relationship between hypoxia and immune regulation in CAD remains unclear. Therefore, it is very important to evaluate the relationship between hypoxia and immunity in CAD for the diagnosis and treatment.

Recently, machine learning algorithms have been used to identify disease biomarkers and therapeutic targets, investigate pathogenesis, and forecast clinical outcomes. Examples include the least absolute shrinkage and selection operator (LASSO), support vector machine recursive feature elimination (SVM-RFE), and random forest (RF) (Wang et al., 2022). Therefore, this study aims to identify potential hypoxia-related genes (HRGs) in CAD. First, The GSE113079 dataset, which was acquired from the Gene Expression Omnibus (GEO) database, underwent bioinformatics studies to identify differentially expressed genes (DEGs) and examined the kinds of immune cell infiltration between CAD and control samples. Then, the DEGs and the HRGs, which were obtained from GSEA MsigDB, were intersected to identify hub genes. In particular, we analyzed the correlation between hub genes and immune infiltrating cells or metabolism-related genes. Furthermore, we constructed the “lncRNA-miRNA-mRNA” ceRNA network. This study may provide new hypoxia-related biomarkers for the diagnosis and treatment of CAD and subsequent fatal events.

## 2 Materials and methods

### 2.1 Data collection and preprocessing

The gene expression profile data was collected from the GEO (http://www.ncbi.nlm.nih.gov/geo/) database with series numbers GSE113079, GSE48166, GSE141512 and GSE56885. The GSE113079 dataset contains 93 CAD patients and 48 healthy controls; the analysis was performed on the GPL20115 platform. The GSE48166 dataset contains 15 ischemic cardiomyopathy patients and 15 healthy controls; the analysis was performed on the GPL9115 platform. The GSE141512 dataset contains 6 myocardial infarction (MI) patients and 6 healthy controls; the analysis was performed on the GPL17586 platform. The GSE56885 dataset contains 4 CAD patients and 2 healthy controls; the analysis was performed on the GPL15207 platform (Table 1). Two hundred hypoxia-related genes (HRGs) and 969 metabolism-related genes were obtained from gene set enrichment analysis (GSEA, https://www.gsea-msigdb.org/gsea/).

**TABLE 1:** The datasets used in the analysis. CAD: Coronary artery disease, HC: health controls, ICM: ischemic cardiomyopathy, MI: Myocardial infarction.

Dataset ID	Tissue	No. of samples	GPL ID	Usage here	References
GSE113079	Blood	93 CAD, 48 HC	GPL20115	Training set	Li et al. (2018)
GSE48166	Heart	15 ICM, 15 HC	GPL9115	validation set	
GSE141512	Blood	6 MI, 6 HC	GPL17586	validation set	Osmak et al. (2020)
GSE56885	Blood	4 CAD, 2 HC	GPL15207	validation set	
GSE131778	Coronary artery	4 CAD	GPL20301	Training set	Wirka et al. (2019)

### 2.2 Identification of hypoxia-related DEGs in CAD

The “limma” R package (version 3.52.4) was used to identify the DEGs and differentially expressed lncRNAs (DElncRNAs) between the CAD and control samples. The threshold value was set to |log2FC| > 0 and FDR <0.05. Genes that crossed over between DEGs and HRGs were subsequently referred to differentially expressed hypoxia-related genes (DE-HRGs). Based on the “ggplot2” (version 3.4.1) program, volcano plots displayed the DEGs and DElncRNAs. The “Venndiagram” (version 1.7.3) software is used to create a Venn diagram that displays the number of DE-HRGs.

### 2.3 GO and KEGG pathway enrichment analysis of DE-HRGs

Gene Ontology (GO) biological process and Kyoto Encyclopedia of Genes and Genomes (KEGG) annotation were performed to analyze the biological functions of the DE-HRGs. Using the R package “clusterProfiler,” (version 4.6.2) GO functional annotation and KEGG pathway enrichment were carried out. The significance threshold for enrichment analysis was set at 0.05. The top 10 results were displayed in the enrichment scatter plots based on the “enrichplot” (version 1.18.3) and “ggplot2” (version 3.4.1) packages.

### 2.4 Construction of the protein–protein interaction network of DE-HRGs

It was done by using the STRING database (http://string-db.org) to examine how the DE-HRGs interacted with each another. Then the protein-protein interaction (PPI) network was built and visualized using Cytoscape software 3.8.0 (http://cytoscape.org/).

### 2.5 Screening hub genes based on machine learning algorithms

We used three machine learning algorithms to screen hub genes for CAD. First, Using the “glmnet” R package (version 4.1.6), the LASSO logistic regression approach was used to identify candidate genes. Then, LASSO classification was performed using binomial distribution variables and a standard error λ value as the minimum standard (1SE standard), and 10 cross-validation variables were selected (Zhao et al., 2022). Next, Support Vector Machines (SVM-REF) were supervised learning models for analyzing data in classification and regression analysis. Given a set of training data marked with two categories, SVM built a model that assigned testing data into one category or the other, making it a non-probabilistic binary linear classifier. Because of SVM’s excellent accuracy, sensitivity and specificity, it is a suitable strategy for predicting continuous variables and generating predictions without significant changes, and it is not limited by the variable environments of Random Forest at the same time. Then, LASSO regression, SVM and RF were used to select the most important hub genes in this study.

Each chosen hub gene’s receiver operating characteristic (ROC) graph was examined to confirm its precision. ROC curve analysis was performed using the “pROC” software package (version 1.18.0), and hub genes with AUC >0.7 were regarded as helpful for disease detection (Wang et al., 2020).

### 2.6 Evaluation of the correlation between hub genes and metabolism-related genes

969 genes involved in metabolism were obtained in total from gene set enrichment analysis (GSEA). Metabolism-related genes that intersect with DEGs were used as differentially expressed metabolism-related genes. The “stats” package (version 4.2.2) was used to conduct a Pearson correlation analysis between hub genes and differentially expressed metabolism-related genes; the heatmap displayed all the outcomes.

### 2.7 Validation of hub genes

Three microarray datasets for CAD (GSE48166, GSE141512, GSE56885) (Table 1) were retrieved from the GEO database for validation of hub genes expression. The “limma” package (version 3.54.2) was also used to identify differential genes with a threshold of |log2FC| > 0 and *p* < 0.05.

### 2.8 Validation of mRNA expression of hypoxia-related genes

Ten whole blood samples from CAD patients were collected from the Second Hospital of Hebei Medical University, 10 whole blood samples from the health check-up population were collected from the Great Wall Physical Examination Center, and total blood RNA from the population was extracted using the RNAprep Pure Hi-Blood Kit.

The mouse cardiomyocyte line HL1 was cultured in MEM containing 10% fetal bovine serum (FBS) at 37°C in a 1% O2 incubator. The coronary artery disease group was given 20 ng/mL TNFα treatment. Total cellular RNA was extracted using TRIpure (Aidlab, CN). The procedure was performed according to the manufacturer’s instructions.

RNA quality and concentration were assessed using a NanoDrop 2000 (Thermo Fisher Scientific, United States). Then, we used a cDNA synthesis kit (Thermo Fisher Scientific, #K1622) to obtaine cDNA by reverse transcription, and analyzed by using qRT‒PCR (Tiangen, FP205). Finally, mRNA expression was normalized to the GAPDH gene.

### 2.9 Gene set enrichment analysis

The hub genes’ potential role was determined using GSEA. The Molecular Signature Database was used to obtain the reference gene set of choice. (MSigDB). The standard for substantial enrichment was *p* < 0.05.

### 2.10 Evaluation of immune cell infiltration and its correlation with hub genes

The CIBERSORT method was utilized to assess the immune cells’ infiltration in the CAD samples from GSE113079. The software program “ggplot2” (version 3.4.1) was used to plot cumulative histograms to show the percentage of 22 immune cell infiltrates in CAD patients after acquiring the expression matrix of immune cells as per the instructions on the CIBERSORT website. Violin diagrams were made to visualize differences in the 22 infiltrating immune cells between the CAD and control groups using the “ggplot2” package. Pearson correlations were calculated between the 22 infiltrating immune cells, and the results were displayed by plot correlation heatmaps using the “corrplot” (version 0.92) software package. Spearman correlations between identified hub genes and infiltrating immune cells were calculated using the “stats” package and then visualized using the “ggplot2” package.

### 2.11 Analysis of single-cell sequencing data

To further analyze the cellular distribution of the screened hub genes in CAD samples, we downloaded the CAD-associated single-cell sequencing dataset GSE131778, which contains 4 CAD samples. The utilities in the Seurat package were used to scale and normalize the gene expression data. The top 2000 highly variable genes were then filtered using the “FindVariableFeatures” tool. Using the “RunPCA” function, principal component analysis (PCA) was also carried out on the single-cell expression matrix limited to the highly variable genes. Cell clustering analysis was performed using the “FindClusters” function. Subsequently, the RunUMAP and RunTSNE functions were used for dimensionality reduction. The type of each cell was determined according to the annotated results provided in the original paper (Wirka et al., 2019).

### 2.12 Construction of the lncRNA–miRNA–mRNA ceRNA network

Coexpression of DElncRNA and hub genes were analyzed by Pearson correlation. Only DElncRNA-hub gene pairs with correlation coefficients >0.5 and *p* < 0.05 were selected. Subsequently, the “mircode” database was used to identify potentially interacting lncRNA-microRNA pairs. The TargetScan database was selected to identify miRNA-mRNA pairs. Finally, a lncRNA-miRNA-mRNA network consisting of four hub genes was constructed.

## 3 Results

### 3.1 Identification of DE-HRGs and DelncRNAs in patients with CAD

**FIGURE 1:**
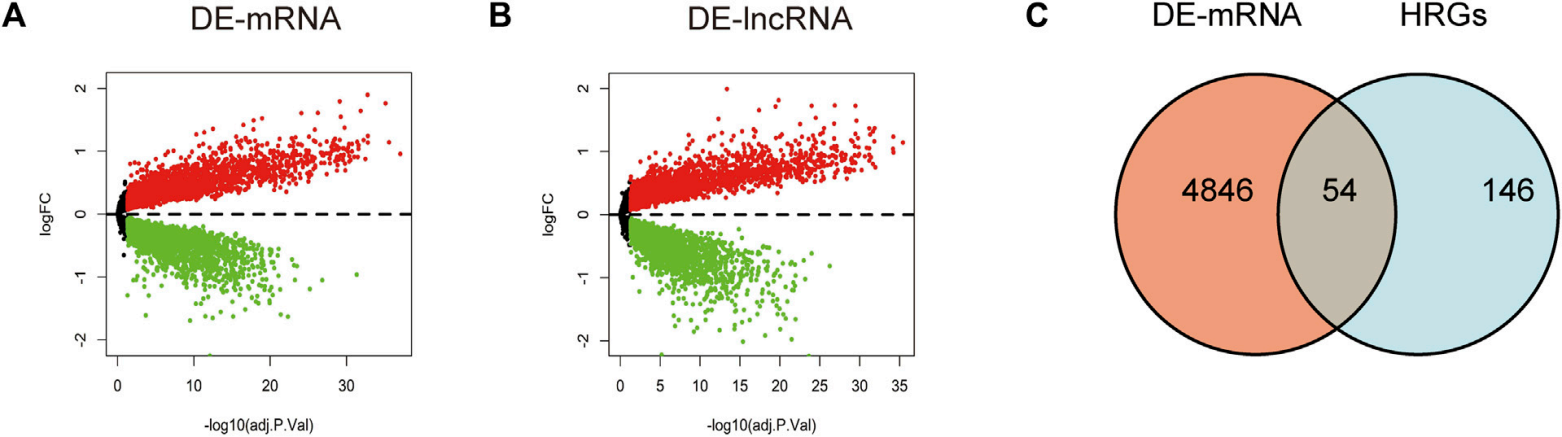
Identification of differentially expressed genes (DE-mRNAs) and differentially expressed long non-coding RNAs (DE-lncRNAs). **(A, B)** Volcano plot showing differentially expressed mRNAs **(A)** and lncRNAs **(B)** in patients with coronary artery disease *versus* the normal control population. Red dots represent upregulated genes and blue dots represent downregulated genes with a threshold of |log2FC| >0 and adjusted to *p* < 0.05. **(C)** Shows DE-mRNAs and hypoxia-related genes taken to intersect to obtain differential hypoxia-related genes (DE-HRGs).

Through differential expression analysis of GSE113079, 4,900 DEGs and 4,766 DElncRNAs were differentially expressed in CAD compared with the control samples, with thresholds of |log2FC| > 0 and adjusted *p* < 0.05 (Figures 1A, B). To investigate the differentially expressed hypoxia-related genes (DE-HRGs) in patients with CAD, we downloaded 200 HRGs from GSEA MSigDB. After taking the intersection of DEGs and HRGs, a total of 54 DE-HRGs were obtained, among which 25 genes were upregulated and 29 genes were downregulated (Table 2; Figure 1C).

**TABLE 2:** 54 DE-HRGs’ differential expressions were analyzed in the GSE113079 datasets.

ID	LogFC	AveExpr	t	*P*. Value	Adj.P.Val	B
ADM	1.177898691	2.639613233	16.91687718	5.71E-36	5.69E-33	71.24674002
AK4	0.602814546	−0.853237677	11.06527353	5.91E-21	1.65E-19	36.98379847
BGN	0.773060954	0.719893874	9.957399096	4.47E-18	8.11E-17	30.41910353
FOSL2	−0.789111529	3.06663182	−9.543590565	5.18E-17	7.72E-16	27.99525002
BRS3	1.110478566	−3.452760102	8.947334419	1.70E-15	2.01E-14	24.54408149
PPFIA4	0.593092808	−1.628856157	8.763000817	4.95E-15	5.43E-14	23.48920287
KDM3A	−0.508844792	0.754002161	−8.597543083	1.28E-14	1.33E-13	22.54793138
COL5A1	0.39433662	−2.958092439	7.712827781	1.90E-12	1.40E-11	17.62129869
VLDLR	0.513949643	−3.72646428	7.665238254	2.47E-12	1.78E-11	17.36212121
ZNF292	−0.370763153	2.590921986	−7.409683041	1.00E-11	6.57E-11	15.98200801
TNFAIP3	−1.106946977	3.026236157	−6.87830132	1.74E-10	9.59E-10	13.18126513
PGM1	0.381,608,887	2.081204243	6.717289189	4.04E-10	2.12E-09	12.35287457
PPP1R15A	−0.915258134	3.08301881	−6.635053993	6.20E-10	3.16E-09	11.93369921
CDKN1B	−0.388402362	3.137457981	−6.465686782	1.48E-09	7.15E-09	11.07905864
GPC1	0.302228389	−0.341871509	6.25357357	4.35E-09	1.96E-08	10.02589327
ISG20	−0.4150531	2.098440901	−6.188293323	6.04E-09	2.66E-08	9.705768249
FAM162A	−0.287739553	−0.231950945	−6.115255069	8.69E-09	3.74E-08	9.349898394
LDHA	−0.33815833	3.252647287	−5.86817191	2.93E-08	1.17E-07	8.164615449
NAGK	0.419358494	4.572588636	5.561814521	1.27E-07	4.69E-07	6.736959945
CCNG2	−0.320543881	−0.497989588	−5.224367568	6.05E-07	2.03E-06	5.222084866
HMOX1	0.428118499	1.289430301	4.934557957	2.20E-06	6.87E-06	3.972754253
GRHPR	0.208960604	1.521280325	4.872382583	2.89E-06	8.87E-06	3.711255043
BNIP3L	−0.445126983	1.307232102	−4.689443816	6.32E-06	1.87E-05	2.955715862
TPBG	0.554799664	−3.08784889	4.652716803	7.38E-06	2.16E-05	2.806574854
PKP1	0.401753519	−3.856222634	4.553825222	1.12E-05	3.18E-05	2.409308824
CAV1	0.514966278	−2.657643106	4.290211391	3.27E-05	8.65E-05	1.381826824
SAP30	−0.371052544	−0.073451892	−4.255632538	3.75E-05	9.84E-05	1.250531678
ENO3	−0.22722186	−1.154129651	−4.210476456	4.48E-05	0.000116476	1.080315804
IL6	−0.909638394	−3.21582843	−4.192033335	4.82E-05	0.000124302	1.011200973
DPYSL4	0.263058085	−2.033101403	4.00560555	9.90E-05	0.00024259	0.325997987
PNRC1	−0.186695873	5.208128173	−3.810543147	0.000205196	0.000481407	−0.364191088
DDIT4	−0.490702596	2.205571939	−3.774292319	0.000234295	0.000544072	−0.489374392
AKAP12	−0.438773726	−3.31947005	−3.719078447	0.00028624	0.000656852	−0.678157081
ATP7A	0.14189323	0.94874353	3.702399409	0.000303963	0.000694325	−0.734734637
MAFF	−0.386872667	−0.488479686	−3.624852195	0.000400885	0.000899735	−0.995022033
VHL	0.392024823	5.670599729	3.589504975	0.000454152	0.001014145	−1.112146126
PDK1	−0.387385851	−0.078648402	−3.575443857	0.000477142	0.001061618	−1.158471957
SULT2B1	0.326995183	−4.240134025	3.435962329	0.000772837	0.001666953	−1.609728949
PRKCA	−0.280966798	−0.370765449	−3.416685145	0.000825198	0.001773175	−1.670902328
PAM	−0.268212621	−0.781255743	−3.321019084	0.001137942	0.002387911	−1.970146405
NOCT	−0.496862247	0.380669063	−3.244267616	0.001465535	0.003023613	−2.204957998
KIF5A	0.233734832	−2.534858673	3.100223556	0.002328731	0.004637683	−2.6328015
PRDX5	−0.288973957	1.421154393	−3.09227385	0.002387945	0.004748491	−2.655921135
ZFP36	0.20174277	6.658195981	3.02444134	0.002952834	0.005785204	−2.851077838
MXI1	−0.162199423	1.382007366	−2.978041764	0.003407631	0.006609853	−2.982377781
PDK3	−0.097595743	2.6291518	−2.622727795	0.009666371	0.017316924	−3.927630933
HK1	−0.054582231	−5.167922069	−2.5990085	0.010327318	0.018414087	−3.986877142
VEGFA	−0.263681718	−4.932100907	−2.573697138	0.0110772	0.019632601	−4.049559837
GAA	0.200306828	0.134587598	2.566478209	0.011299849	0.019996092	−4.067334834
ATF3	0.307700421	0.932697695	2.531895124	0.012423135	0.021804763	−4.151855918
MT2A	−0.290126487	0.843244915	−2.476297183	0.014439407	0.025002378	−4.285537754
GAPDH	0.126840398	7.102301648	2.458303882	0.015151763	0.026144991	−4.328218456
TIPARP	−0.180224942	−0.751647504	−2.397778697	0.017782872	0.030246438	−4.469683887
KDELR3	0.158662234	−1.470976755	2.202095	0.02925745	0.047795162	−4.904674271

### 3.2 Functional enrichment analysis of DE-HRGs

**FIGURE 2:**
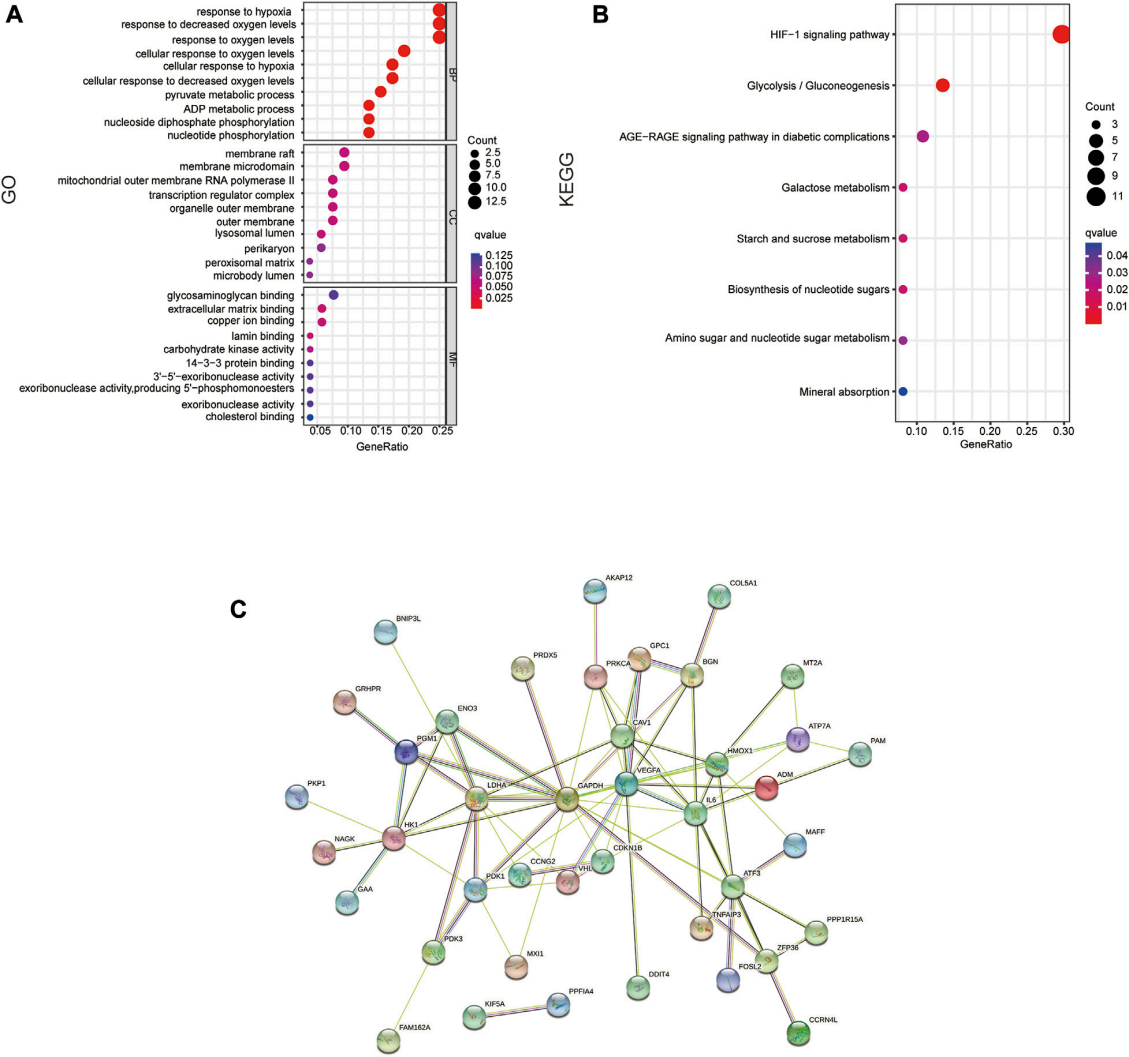
Functional annotation of DE-HRGs. **(A)** GO enrichment analysis of the DE-HRGs. **(B)** KEGG enrichment analysis of the DE-HRGs. **(C)** Protein–protein interaction (PPI) network of the DE-HRGs.

On 54 DE-HRGs, GO and KEGG pathway studies were conducted to investigate putative biological roles and pathways. The enrichment scatter plot present the top 10 findings. GO analysis revealed that the pathways for hypoxia, metabolism, membrane microregions, and outer mitochondrial membrane were considerably enriched in DE-HRGs (Figure 2A). The hypoxia-inducible factor HIF1 signaling route, the AGE-RAGE signaling pathway, metabolism, and other associated pathways were all engaged in these DE-HRGs, according to KEGG pathway analysis (Figure 2B).

To further investigate the protein interactions of the 54 DE-HRGs, we constructed a PPI network. We submitted all DE-HRGs to the STING database. Then, the obtained results were visualized by Cytoscape software, which presented the network interaction among these genes. After removing the separated DE-HRGs, 54 nodes and 83 edges were included (Figure 2C).

### 3.3 Screening for hub signature genes

**FIGURE 3:**
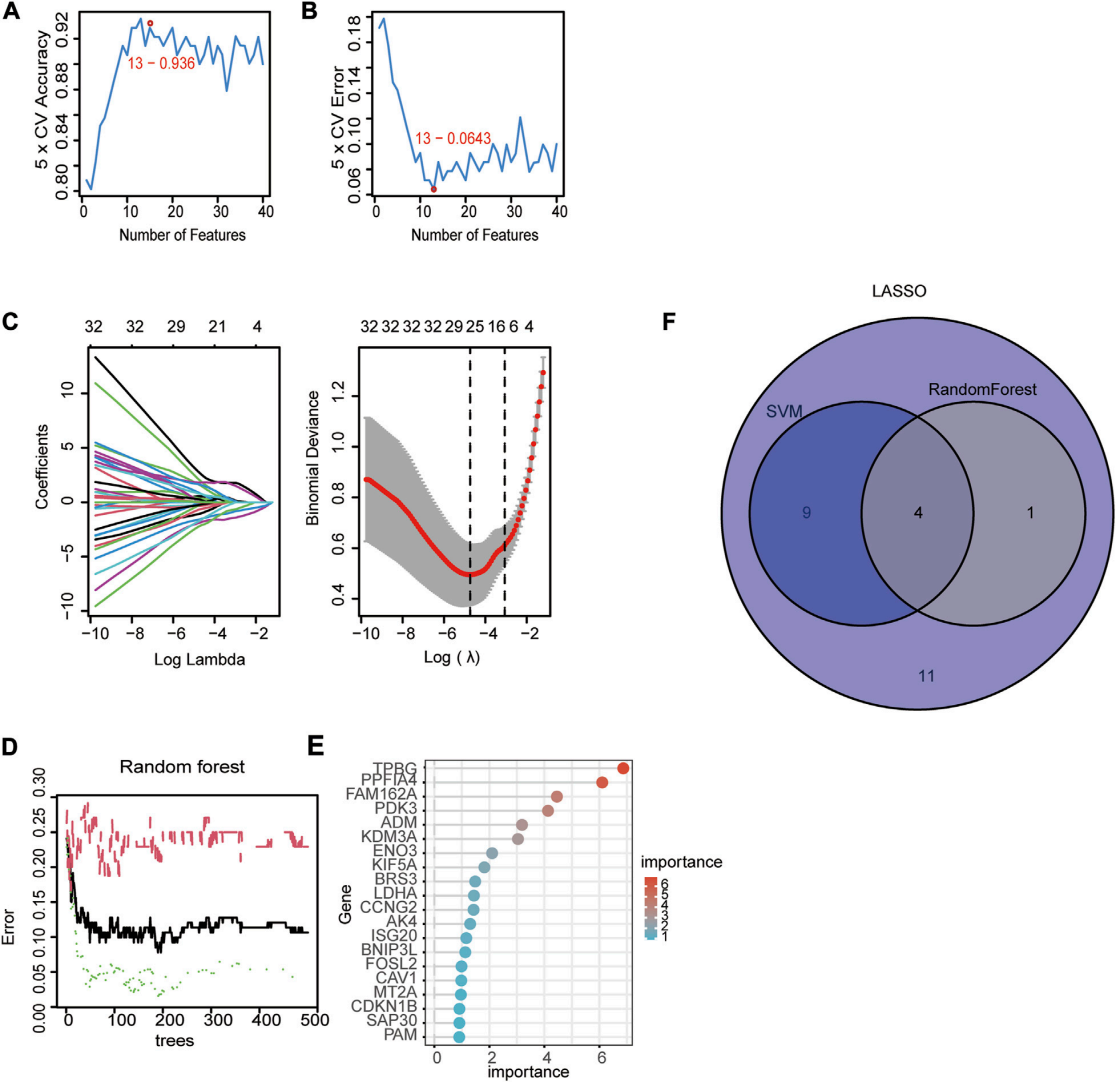
Identification of diagnostic genes using three machine learning algorithms. **(A, B)** Based on support vector machine‐recursive feature elimination (SVM-RFE) to screen hub genes. **(C)** Least absolute shrinkage and selection operator (LASSO) regression algorithm to screen hub genes. **(D)** Random forest (RF) algorithm to screen hub genes. **(E)** The rank of genes in accordance with their relative importance. **(F)** Venn diagram of hub genes in three machine learning algorithms (SVM, LASSSO, RF).

We used three machine algorithms to identify hub signature genes. For the SVM-RFE algorithm, 13 genes were selected when classifier error was minimized, which contained FAM162A, KIF5A, BGN, TPBG, AKAP12, PPFIA4, TIPARP, KDELR3, FOSL2, CCNG2, NAGK, ADM and DDIT4 (Figure 3A, B. For the LASSO algorithm, we chose the minimum criteria for building the LASSO classifier due to higher accuracy, and 25 signature genes were identified, including ADM, BGN, FOSL2, BRS3, PPFIA4, KDM3A, VLDLR, PGM1, PPP1R15A, FAM162A, NAGK, CCNG2, TPBG, SAP30, IL6, DPYSL4, DDIT4, AKAP12, VHL, KIF5A, PDK3, VEGFA, MT2A, TIPARP, and KDELR3 (Figure 3C). For the random forest algorithm, the top 5 important genes were selected: TPBG, PPFIA4, FAM162A, PDK3, and ADM (Figure 3D, E). Four overlapping hub genes (ADM, PPFIA4, FAM162A, and TPBG) were identified by the intersection of the abovementioned three algorithms (Figure 3F).

### 3.4 Expression analysis and diagnostic efficacy of hub genes for CAD

**FIGURE 4:**
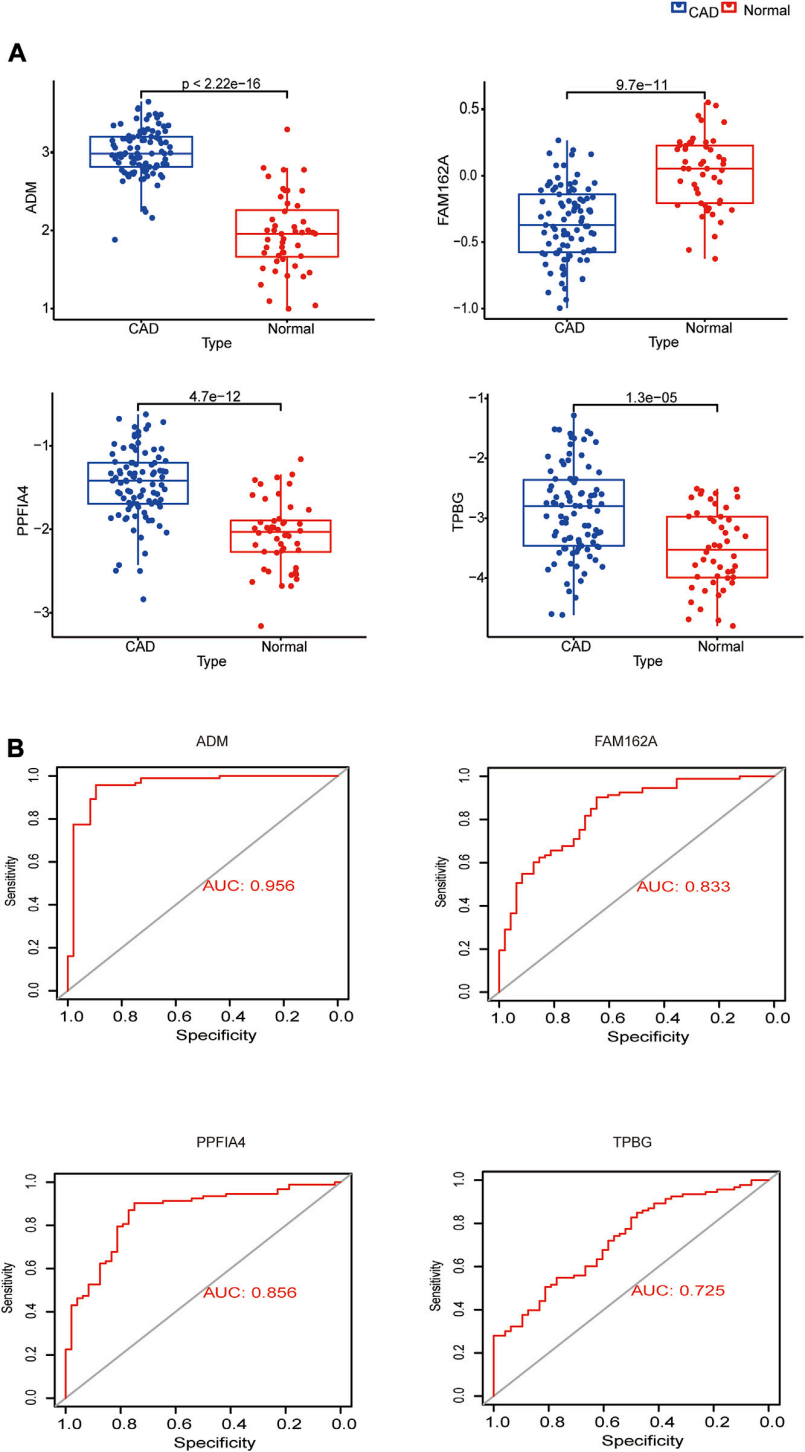
Expression analysis and diagnostic efficacy of hub genes in the prediction of CAD. **(A)** Box plots showing the mRNA expression of hub genes in CAD patients and Normal control in the GSE113079 dataset. **(B)** ROC curves estimating the diagnostic performance of hub genes.

ADM, PPFIA4, and TPBG exhibited greater expression levels in the CAD group than in the control group, but FAM162A showed lower expression levels in the CAD group, when we compared the expression of these genes between CAD and control samples in the GSE113079 dataset (Figure 4A). We mapped ROC curves to investigate the efficacy of the 4 hub genes as CAD diagnostic biomarkers (Figure 4B). The AUC values for the 4 hub genes were >0.7, indicating high diagnostic performance. In the four hub genes, ADM’s AUC was the highest at 0.956, which is significant.

### 3.5 Identification of correlations between hub genes and metabolism-related genes

**FIGURE 5:**
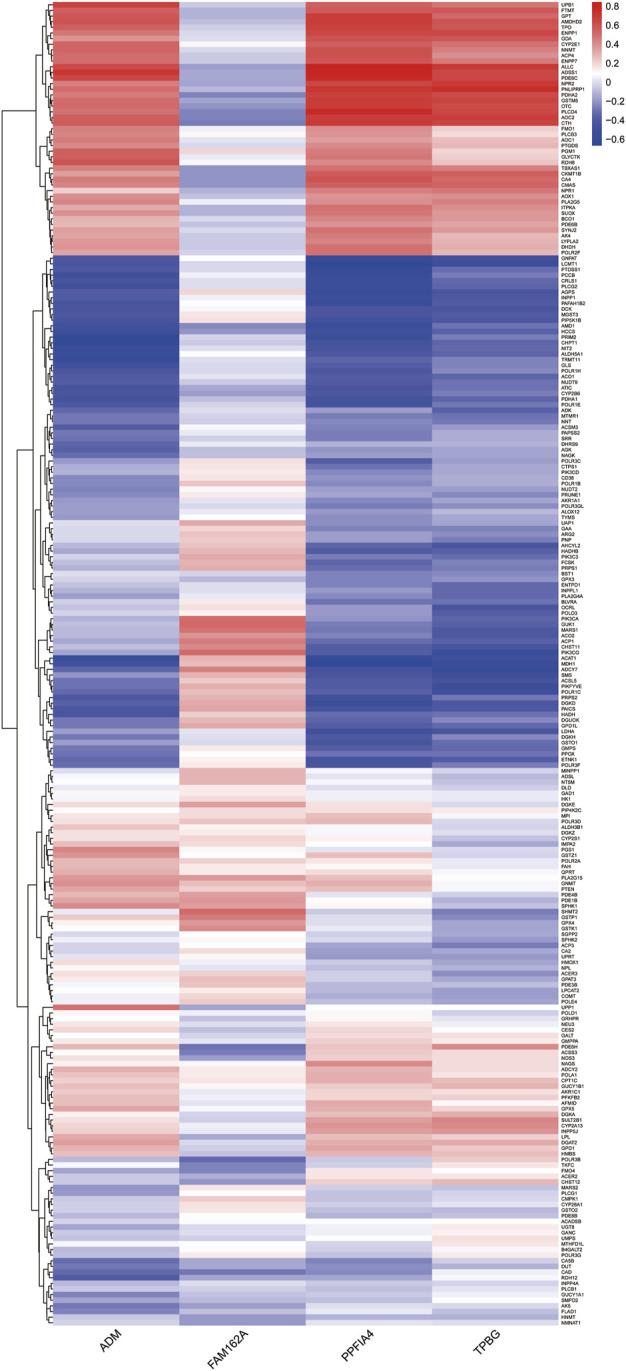
Heatmap indicating the correlation between the hub genes and differentially expressed metabolism-related genes. The color represents the *p*-value of the correlation, with positive correlations in red and negative correlations in blue.

Since pathway analysis showed that DE-HRGs were enriched in metabolism-related pathways, we analyzed the relationship between hub genes and metabolism-related genes. Among 969 metabolism-related genes, there were 235 differentially expressed genes between CAD and control samples in the GSE113079 dataset, and Pearson correlation analysis showed that most differential metabolism-related genes were correlated with hub genes, either positively or negatively (|r| ≥ 0.3, *p* < 0.05). Notably, three upregulated genes (ADM, PPFIA4, TPBG) were more strongly correlated with differentially expressed metabolism-related genes (Figure 5; Supplementary Table S1).

### 3.6 External validation of 4 hub genes for CAD

To verify whether the 4 hub genes are differentially expressed in other CAD datasets, we selected three additional microarray datasets (GSE48166, GSE141512 and GSE56885) for external validation. Of the 4 hub genes, ADM was upregulated in CAD (GSE56885), PPFIA4 and TPBG were upregulated in patients with ischemic heart disease (GSE48166), and FAM162A was downregulated in patients with myocardial infarction (GSE141512) (Table 3).

**TABLE 3:** Hub gene logFC value in CAD datasets (*p* < 0.05).

	GSE48166	GSE141512	GSE56885
ADM			1.423750038
PPFIA4	0.503695578		
TPBG	0.682006896		
FAM162A		−0.205765639	

To further confirm the expression of hypoxia-associated genes, we performed qRT‒PCR experiments in HL1 cells and human whole blood samples.

**FIGURE 6:**
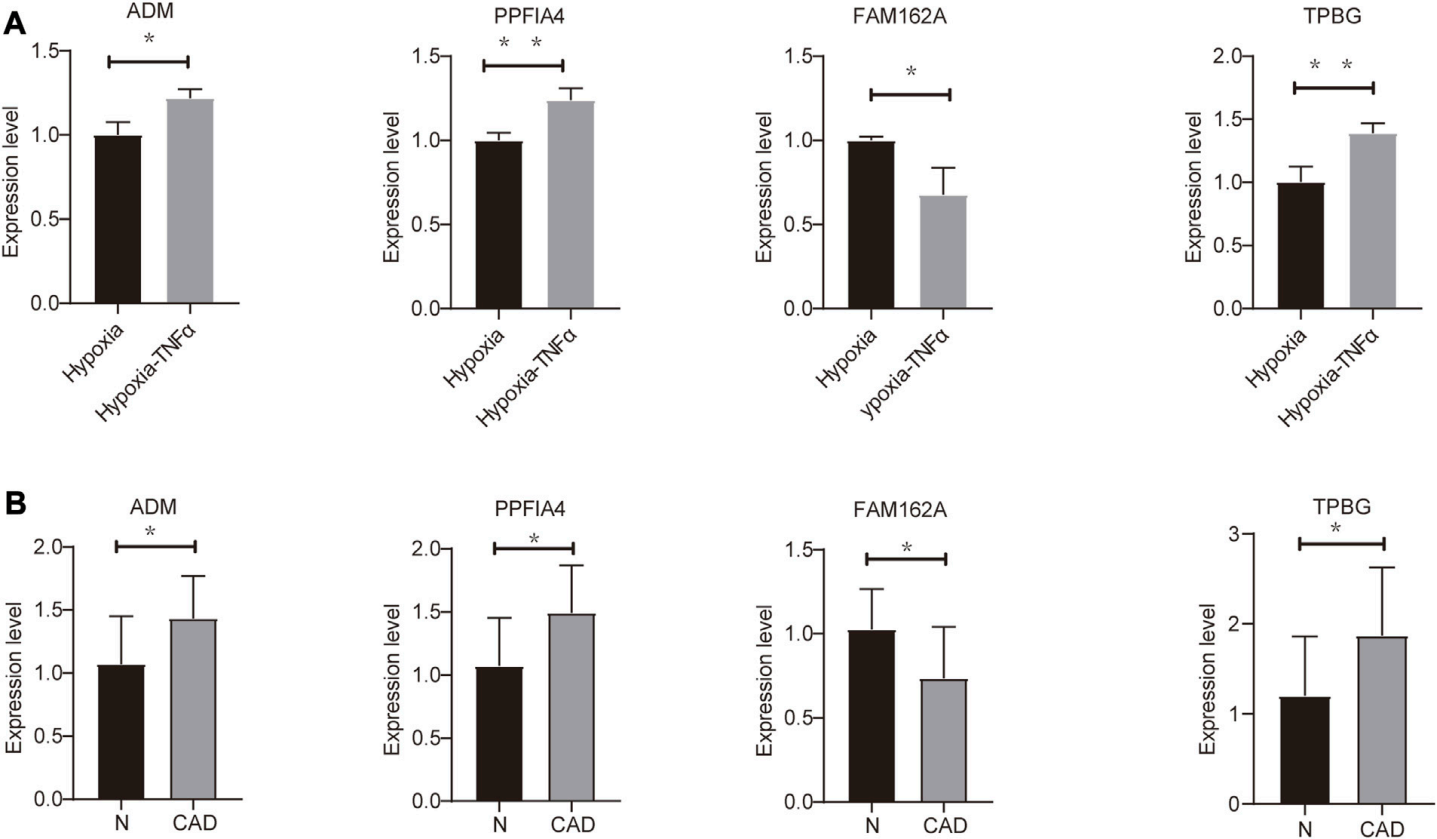
Validation of mRNA expression of hypoxia-related genes **(A)** Differential expression of hub genes in hypoxic control (hypoxia) and hypoxic coronary artery disease groups (Hypoxia-TNFα). **(B)** Differential expression of hub genes in 10 pairs of CAD patients and healthy physical examination population (N). *p < 0.05 and **p < 0.01.

To assess the expression of four hypoxia-related hub genes (ADM, PPFIA4, FAM162A and TPBG) between CAD patients and the control population, qRT‒PCR was used to quantify mRNA expression levels. Compared to the control population, ADM, PPFIA4 and TPBG expression was upregulated, and FAM162A expression was downregulated in the whole blood of CAD patients. In addition, we confirmed the mRNA levels of these hub genes in mouse HL1 cells. Compared to the hypoxic cultured HL1 cells, ADM, PPFIA4 and TPBG expression were upregulated, and FAM162A expression was downregulated in TNFα-treated hypoxic HL1 cells (Figure 6A, B). All primer sequences were shown in Supplementary Table S2.

### 3.7 GSEA identifies 4 hub gene-associated signaling pathways

**FIGURE 7:**
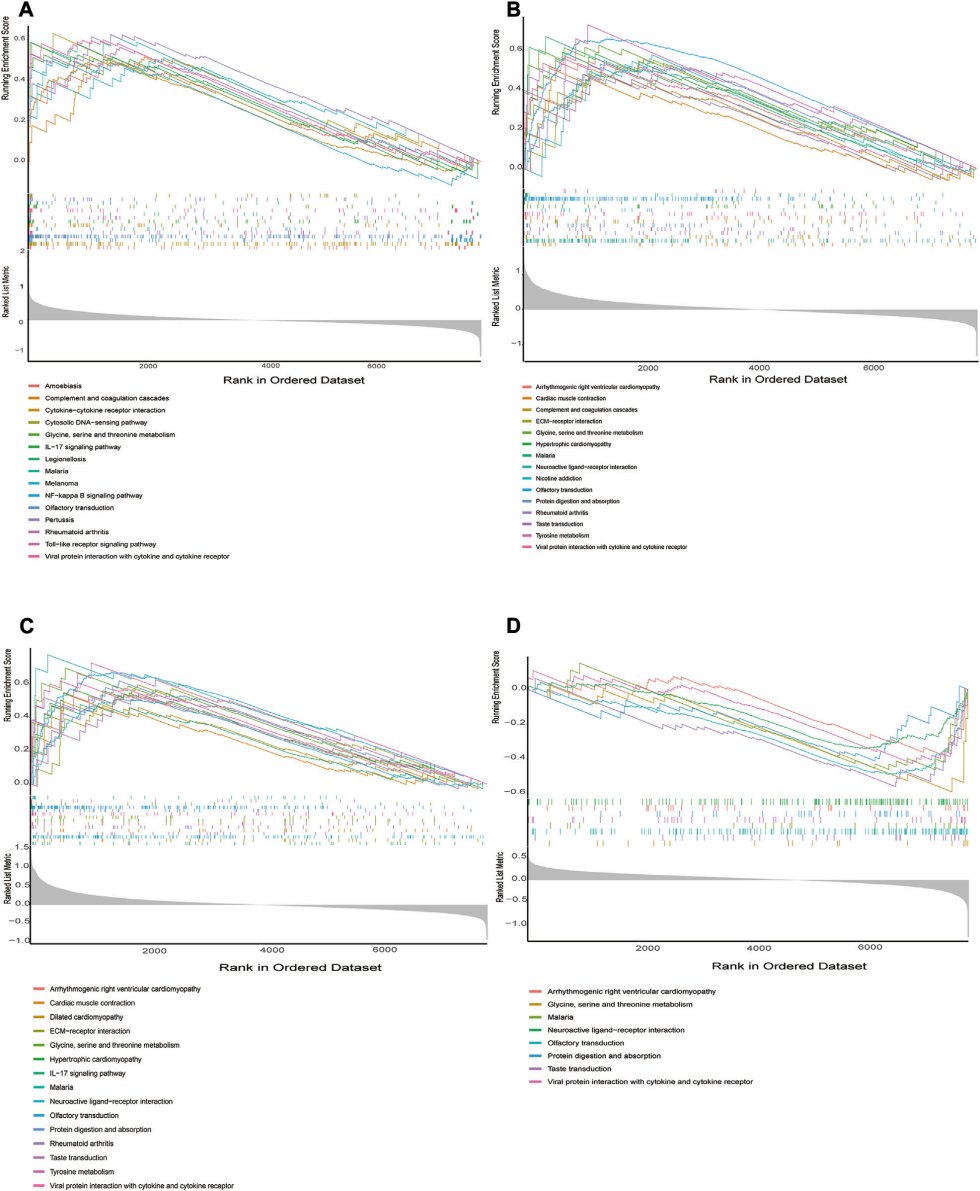
GSEA identifies signaling pathways involved in the hub genes. **(A–D)** The main signaling pathways that are significantly enriched in high or low expressions of characteristic genes. **(A)** ADM, **(B)** PPFIA4, **(C)** TPBG, **(D)** FAM162A.

We performed in GSEA to further illustrate the role of the 4 hub genes. The obtained results demonstrated that ADM, PPFIA4, FAM162A, and TPBG were linked to immune responses (Toll-like receptor signaling pathway, cytokine–cytokine receptor interaction, TIL-17 signaling pathway, NF-kappa B signaling pathway, etc.) in addition to metabolic signaling pathways (Tyrosine metabolism, Glycine, serine and threonine metabolism) (Figure 7A–D).

### 3.8 Analysis of immune cell infiltration

**FIGURE 8:**
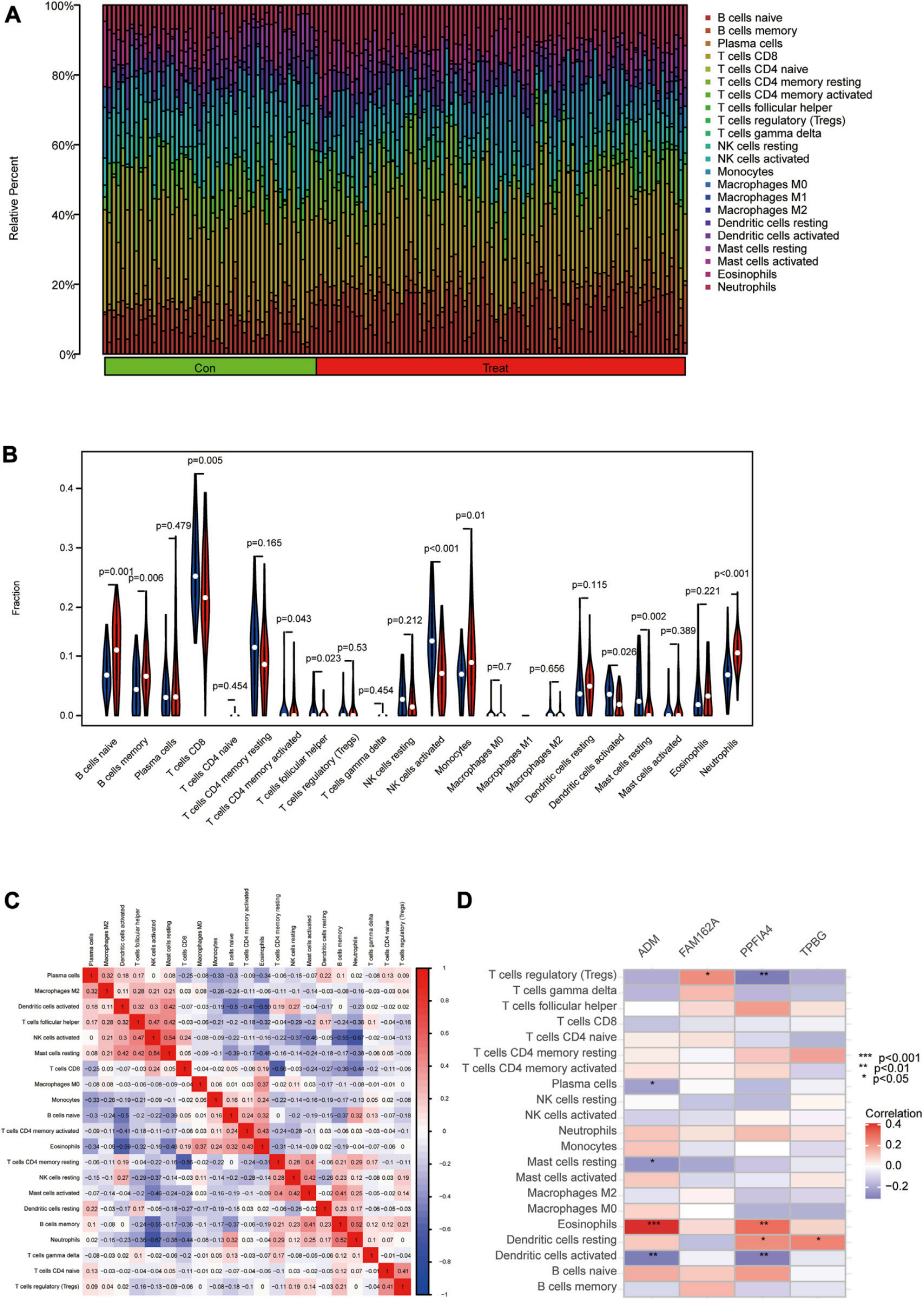
Analyzing and showing immune cell infiltration. **(A)** Immune cell kinds and ratios in patients with CAD. **(B)** A box plot showing the expression of 22 different immune cell types between CAD and controls. **(C)** A heat map demonstrating correlation for 21 different immune cell types. The degree of the correlation is shown by the size of the colored squares; red indicates a positive correlation, and blue indicates a negative correlation. The association is greater the darker the hue. **(D)** Correlation between immune cells and important genes.

The association of immune cell infiltration between CAD and control samples was further investigated in the GSE113079 dataset using the CIBERSORT algorithm. The proportions of 22 immune cell subtypes in each sample were shown in Figure 8A. The obtained results showed that CD8 T cells were significantly enriched, and macrophage M1 expression was deficient in both CAD and controls. As shown in Figure 8B and ten immune cell types were significantly different between the CAD and control groups (*p* < 0.05), including naive B cells, memory B cells, CD8 T cells, activated memory CD4 T cells, follicular helper T cells, activated NK cells, monocytes, activated dendritic cells, resting mast cells and neutrophils.

Furthermore, we used Pearson correlation to estimate the correlation between the 24 immune cells. The obtained results revealed positive correlations between activated NK cells and resting mast cells, memory B cells and neutrophils. Meanwhile, negative correlations were found between activated dendritic cells and naive B cells or eosinophils, activated NK cells and memory B cells or neutrophils, CD8 T cells and resting memory CD4 T cells (Figure 8C). We then used Spearman correlation analysis to estimate the correlation between the 4 hub genes and immune cells. ADM was positively correlated with eosinophils (r = 0.41) but negatively correlated with activated dendritic cells (r = −0.31), resting mast cells (r = −0.25), and plasma cells (r = −0.22). FAM162A was positively correlated with regulatory T cells (r = 0.23). PPFIA4 was positively correlated with eosinophils (r = 0.30) and resting dendritic cells (r = 0.23) but negatively correlated with activated dendritic cells (r = −0.30) and regulatory T cells (r = −0.31). TPBG was positively correlated with resting dendritic cells (r = 0.25) (Figure 8D; Supplementary Figure S1).

### 3.9 Single-cell RNA sequencing analysis

**FIGURE 9:**
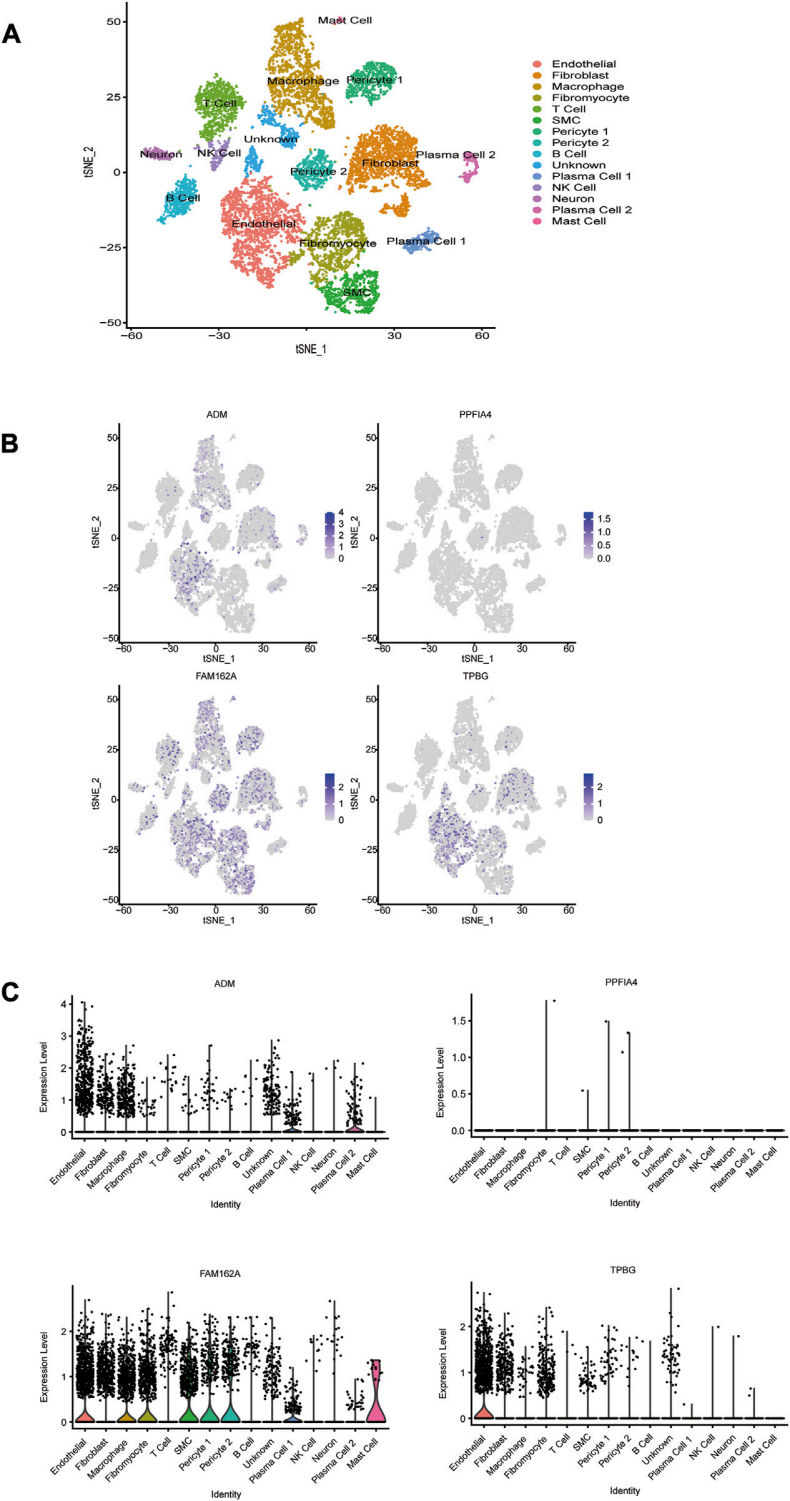
The cell distribution of 4 hub HRGs in CAD. **(A)** t-distribution random neighbor embedding (t-SNE) plot showing annotation and color coding of CAD cell types. The single-cell data were downscaled by the TSNE algorithm to obtain 2 dimensions, tSNE_1 and tSNE_2. **(B)** Scatter plot and **(C)** violin plot show the distribution of 4 hub HRGs in CAD.

To evaluate the expression levels of the 4 hub genes at the single-cell level, CAD-associated single-cell sequencing data from GSE131778 were screened, which contained 4 CAD samples. The t-SNE method was used for manual annotation of clusters, and a total of 15 cell subtypes were identified, including endothelial, fibroblast, macrophage, fibromyocyte, T cell, SMC, pericyte 1, pericyte 2, B cell, plasma cell 1, NK cell, neuron, plasma cell 2, mast cell, and unknown (Figure 9A). The obtained results showed that ADM was mainly expressed in endothelial cells, plasma cells 1 and plasma cells 2. FAM162A was mainly expressed in endothelial cells, macrophages, fibromyocytes, SMCs, pericytes1, pericytes2, plasma cells 1 and mast cells. TPBG was mainly expressed in endothelial cells. However, PPFIA4 was not captured in these cells.

### 3.10 Construction of the lncRNA-miRNA-mRNA ceRNA network

**FIGURE 10:**
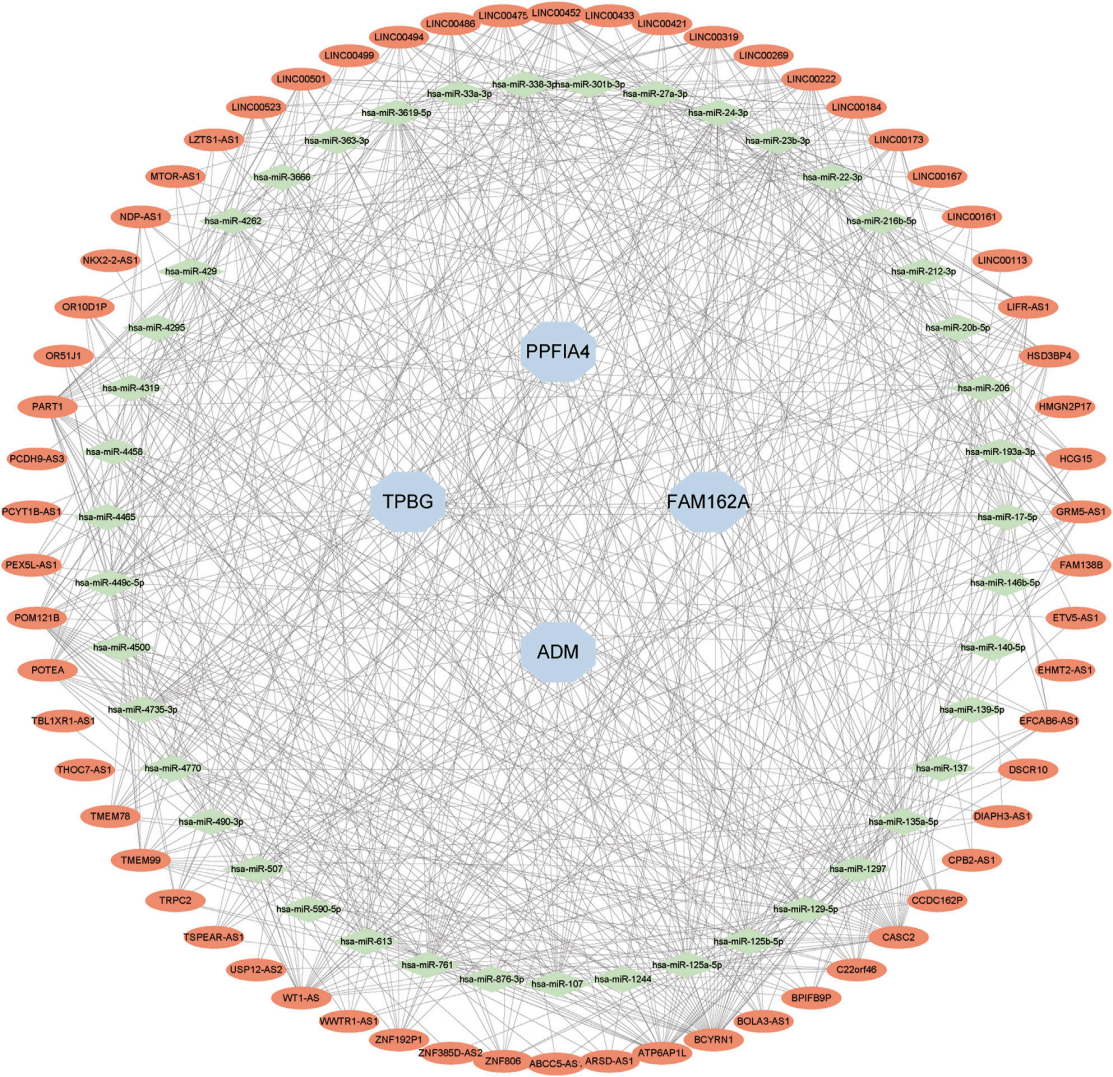
Developing the ceRNA Network. The expected lncRNAs were shown by red nodes. The expected miRNAs were shown by green nodes. Hub genes were represented by blue nodes.

We created a lncRNA-miRNA-mRNA competition endogenous RNA (ceRNA) network to investigate the role of lncRNAs as miRNA sponges in CAD based on the endogenous RNA competition hypothesis. The projected miRNAs were merged with co-expressed upregulated lncRNAs and hub genes into the upregulated ceRNA network. There were 4 hub genes, 43 miRNAs, and 62 lncRNAs in the ceRNA network (Figure 10).

## 4 Discussion

Multiple reports have shown that hypoxia plays a central role in the pathogenesis and pathophysiology of CAD (Yu et al., 2022). However, due to the multiple roles of hypoxia, the mechanism through which hypoxia initiates the development and progression of CAD has not been clarified and needs to be further explored. In this study, we identified hypoxia-related genes and associated pathways in GSE113079 and further validated them in the GSE48166, GSE141512 and GSE56885 datasets.

Herein, we first identified 54 DE-HRGs, and then 4 hub signature genes (ADM, PPFIA4, FAM162A, and TPBG) were screened by three machine algorithms (LASSO regression, SVM-RFE and random forest), which showed reliable diagnostic power for CAD. The 4 hub genes were also validated in other microarray datasets (GSE48166, GSE141512 and GSE56885). Organ protection, anti-inflammatory properties, and tissue repair are just a few of the many different actions and functions of the endogenous vasodilatory peptide that known as adrenalmedullin. Also, there is compelling evidence linking increased plasma and tissue levels of ADM with cardiovascular disease (Yanagawa and Nagaya, 2007; Lucero Garcia Rojas et al., 2021). Several studies have been previously reported to be associated with coronary artery function and cardiovascular outcomes (Nishida et al., 2001; Haberka et al., 2019; Theuerle et al., 2019). PTPRF Interacting Protein Alpha 4 (PPFIA4), which belongs to the PPFIA family, possesses different physiological functions in humans. A previous study discovered that variants of PPFIA4 were associated with supernormal coronary arteries and atrial fibrillation (Low et al., 2017; Kim et al., 2022). FAM162A may serve as possible therapeutic and diagnostic targets for human dilated and ischemic cardiomyopathies because it was differentially expressed at the transcriptomic and proteomic levels in a number of rodent and human heart disease models (Lee et al., 2020). Recently, it was found that Trophoblast Glycoprotein (TPBG) was expressed in adventitial pericyte-like cells (APCs) *in situ* and after *in vitro* expansion and was essential for migration and proangiogenic activities (Spencer et al., 2019). Moreover, our results showed that PPFIA4 and TPBG were significantly involved in CAD, including our 5 recurrent CAD samples and 5 normal samples. ADM, PPFIA4, FAM162A, and TPBG, which were all reported to be hypoxia-associated signature genes, have been less explored in CAD.

In this study, vitro experiments showed that the relative mRNA levels of ADM, PPFIA4, FAM162A, and TPBG were increased in the IH groups compared with the control groups, which revealed that the relationship between the 4 hub genes and hypoxia was related to CAD (Sun et al., 2022).

Both GO enrichment analysis and KEGG analyses suggest that DE-HRGs are associated with hypoxic and metabolic pathways. Abnormal metabolism is a very common feature in CAD, and modulation of related metabolism-associated genes may represent a novel therapeutic approach to treat CAD (Fan et al., 2016). In addition, hypoxic signaling and metabolism changes, such as the glycolytic pathway and fatty acid β-oxidation pathway, are highly interlinked (Wong et al., 2017; Lucero Garcia Rojas et al., 2021). However, the mechanism of adaptive cardiac metabolism under chronic hypoxia remains to be fully characterized (Su et al., 2021). In our previous studies, an ADM antagonist played pivotal roles in metabolic regulation (Liu et al., 2017). Notably, PPFIA4 and FAM162A were also both glycolysis-related genes (Huang et al., 2021; Liu et al., 2022). TPBG (trophoblast glycoprotein, HGNC: 12004), a heavily N-glycosylated transmembrane protein, is a glucose metabolism-related biomarker gene (Zhang et al., 2021). In this study, we found that three upregulated hub genes (ADM, PPFIA4, TPBG) were strongly correlated with differentially expressed metabolic genes in CAD.

To further illustrate the role of hub genes in CAD, we performed GSEA of hub genes. The obtained results showed that the 4 hub genes were mainly enriched in many immune-related biological processes and pathways. It is currently believed that immune cell infiltration plays an important role in the onset and development of CAD (Yang and Xu, 2021). By immune cell infiltration analysis, we found 10 immune cell types that were significantly different between the CAD and control groups, especially CD8 T cells, which were apparently essential in cardiovascular disease. Studies have reported that hypoxia signaling plays a critical role in the initiation or regulation of inflammation in cardiovascular disease (Abe et al., 2017). Furthermore, several *in vitro* studies have demonstrated that ADM and TPBG exert anti-inflammatory effects in cardiovascular disease (Shindo et al., 2019; Spencer et al., 2019). In this study, we speculated that ADM and PPFIA4 may influence CAD by regulating the activity of eosinophils and that PPFIA4 and TPBG may influence CAD by regulating the activity of resting dendritic cells. However, FAM162A and PPFIA4 may play the opposite roles in regulatory T cells. Direct evidence of these associations was not mentioned in previous studies. Further experimental research is needed for classification.

Single-cell sequencing is a ground-breaking approach that enables us to better understand the biological diversity of cells and assess gene expression in individual cells (Song et al., 2022). To screen which cell types the 4 hub hypoxia-related genes are mainly expressed in, we performed single-cell sequencing analysis. We compared the expression of 4 hub genes in 15 cell subtypes in CAD coronary lesions and found that ATM, FAM162A and TPBG were all expressed at higher levels in endothelial cells. It is worth noting that PPFIA4 was not captured in these cells. The functional influence of the 4 hub hypoxia-related genes in these cells on plaques and their relevance *in vivo* are worth further exploration.

As far as we know, this is the first study to report the molecular and immune characteristics associated with hypoxia-related genes in CAD. Our study provides new insights into hypoxia in CAD, thereby providing more evidence for CAD prevention and treatment. However, this study also has several limitations. First, the sample size included is relatively small. Second, the potential molecular mechanisms of hypoxia in CAD, including immune infiltration and metabolism, need to be further explored.

In conclusion, we identified and validated 4 hub hypoxia-related genes that can be used as diagnostic markers for CAD by comprehensive analysis. The 4 hub hypoxia-related genes may influence CAD progression through immune cell infiltration and metabolism. This study provides new insights into hypoxia and CAD.

## Data Availability

Data available on request from the authors.
