# Supplementary material for: Identification and validation of potential hypoxia-related genes associated with coronary artery disease
Source: Front Physiol. 2023 Aug 10;14:1181510. doi: 10.3389/fphys.2023.1181510 (PMC10447898; doi:10.3389/fphys.2023.1181510)
Supplement: Supplementary file 1 [file Table1.DOCX]

Supplementary Material

**Identification and validation of Potential Hypoxia-Related Genes Associated With Coronary artery disease**

Yuqing Jin

*** Correspondence:**

Lianguo Hou: houlianguo@qq.com

Lei Yang: leiyang1127@hotmail.com

# Supplementary Figures and Tables

## Supplementary Figures


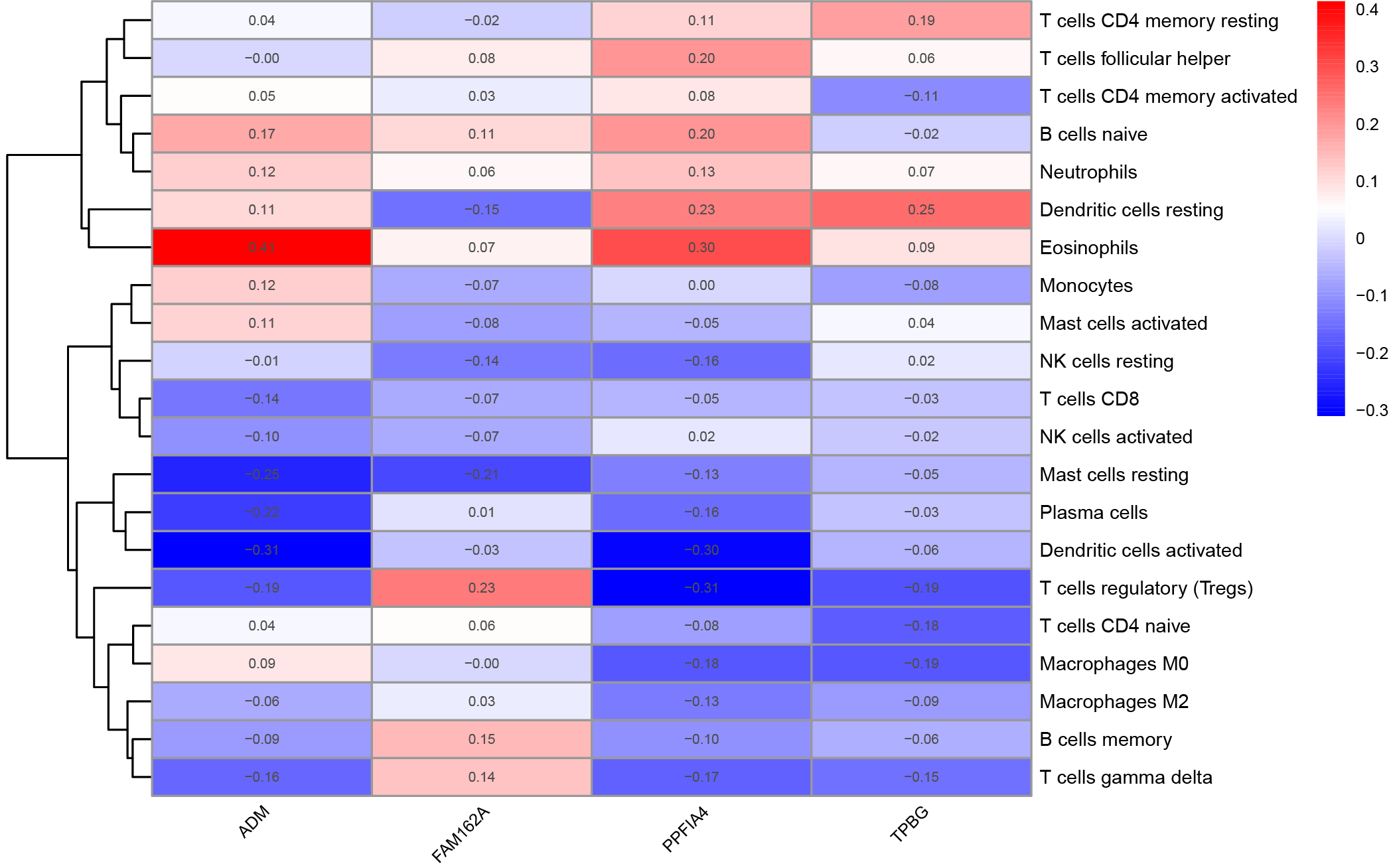


**Supplementary Figure 1.** Correlations between the key genes and immune cells

## Supplementary Table 1

**Supplementary Table 1.** Pearson correlation analysis of most differential metabolism-related genes and hub genes

| Data1 | Data2 | rho | pvalue | relation |
| --- | --- | --- | --- | --- |
| ACAT1 | ADM | 0.56139893 | 4.80156E09 | negative |
| ACO1 | ADM | 0.384783848 | 0.000139952 | negative |
| ACSL5 | ADM | 0.300378056 | 0.003439702 | negative |
| ACSM3 | ADM | 0.368636254 | 0.000277071 | negative |
| ADCY7 | ADM | 0.341721283 | 0.000801171 | negative |
| ADK | ADM | 0.321464437 | 0.001677956 | negative |
| AGK | ADM | 0.358824989 | 0.000412474 | negative |
| AGPS | ADM | 0.377049685 | 0.000194972 | negative |
| ALDH5A1 | ADM | 0.562228983 | 4.50661E09 | negative |
| AMD1 | ADM | 0.478591441 | 1.22021E06 | negative |
| ATIC | ADM | 0.448534676 | 6.51411E06 | negative |
| CA5B | ADM | 0.312238644 | 0.002311689 | negative |
| CAD | ADM | 0.320674222 | 0.001725319 | negative |
| CHPT1 | ADM | 0.610966557 | 7.84386E11 | negative |
| ACAT1 | PPFIA4 | 0.56789437 | 2.91001E09 | negative |
| ACO1 | PPFIA4 | 0.36845693 | 0.000279125 | negative |
| ACP1 | PPFIA4 | 0.316750265 | 0.001978904 | negative |
| ACSL5 | PPFIA4 | 0.383975295 | 0.000144944 | negative |
| ADCY7 | PPFIA4 | 0.459223474 | 3.65625E06 | negative |
| AGPS | PPFIA4 | 0.539624874 | 2.38443E08 | negative |
| AHCYL2 | PPFIA4 | 0.343463631 | 0.000750047 | negative |
| ALDH5A1 | PPFIA4 | 0.406612017 | 5.24613E05 | negative |
| AMD1 | PPFIA4 | 0.551691165 | 9.95039E09 | negative |
| ATIC | PPFIA4 | 0.431634721 | 1.5616E05 | negative |
| CHPT1 | PPFIA4 | 0.461226935 | 3.27398E06 | negative |
| CHST11 | PPFIA4 | 0.378225828 | 0.000185483 | negative |
| ACAT1 | TPBG | 0.491596736 | 5.61862E07 | negative |
| ACO1 | TPBG | 0.338626739 | 0.000899873 | negative |
| ACO2 | TPBG | 0.396498541 | 8.33625E05 | negative |
| ACP1 | TPBG | 0.354813727 | 0.000483586 | negative |
| ACSL5 | TPBG | 0.466302057 | 2.46728E06 | negative |
| ADCY7 | TPBG | 0.586895135 | 6.30772E10 | negative |
| ADK | TPBG | 0.350933968 | 0.000562899 | negative |
| AGPS | TPBG | 0.438630787 | 1.09353E05 | negative |
| AHCYL2 | TPBG | 0.433723713 | 1.40515E05 | negative |
| ALDH5A1 | TPBG | 0.344159145 | 0.000730485 | negative |
| AMD1 | TPBG | 0.30214782 | 0.003244972 | negative |
| CHPT1 | TPBG | 0.344005341 | 0.000734771 | negative |
| CHST11 | TPBG | 0.451103605 | 5.67993E06 | negative |
| CRLS1 | ADM | 0.444470022 | 8.07306E06 | negative |
| CYP2B6 | ADM | 0.428571308 | 1.82078E05 | negative |
| DCK | ADM | 0.441315989 | 9.51758E06 | negative |
| DGKD | ADM | 0.363301728 | 0.000344533 | negative |
| DHRS9 | ADM | 0.339689103 | 0.000864804 | negative |
| ETNK1 | ADM | 0.331695075 | 0.001162361 | negative |
| GLS | ADM | 0.492477744 | 5.32485E07 | negative |
| GNPAT | ADM | 0.420396317 | 2.72329E05 | negative |
| CRLS1 | PPFIA4 | 0.526284129 | 6.02605E08 | negative |
| CYP2B6 | PPFIA4 | 0.405856376 | 5.43365E05 | negative |
| DCK | PPFIA4 | 0.434993949 | 1.31734E05 | negative |
| DGKD | PPFIA4 | 0.516966365 | 1.12499E07 | negative |
| DGKH | PPFIA4 | 0.405684017 | 5.47729E05 | negative |
| DGUOK | PPFIA4 | 0.336924334 | 0.000958781 | negative |
| ETNK1 | PPFIA4 | 0.447732607 | 6.79732E06 | negative |
| FCSK | PPFIA4 | 0.342185279 | 0.000787255 | negative |
| GLS | PPFIA4 | 0.380150947 | 0.000170869 | negative |
| GMPS | PPFIA4 | 0.366299175 | 0.000304968 | negative |
| GNPAT | PPFIA4 | 0.577396688 | 1.37126E09 | negative |
| GPD1L | PPFIA4 | 0.366963889 | 0.000296781 | negative |
| CRLS1 | TPBG | 0.328365659 | 0.001311682 | negative |
| DCK | TPBG | 0.440024501 | 1.01764E05 | negative |
| DGKD | TPBG | 0.420815703 | 2.66829E05 | negative |
| ENTPD1 | TPBG | 0.328243052 | 0.001317499 | negative |
| ETNK1 | TPBG | 0.375185927 | 0.00021093 | negative |
| GMPS | TPBG | 0.324357602 | 0.001514414 | negative |
| GNPAT | TPBG | 0.495115858 | 4.52981E07 | negative |
| GPD1L | TPBG | 0.330753359 | 0.001202946 | negative |
| POLR3F | ADM | 0.317201216 | 0.001948142 | negative |
| PPOX | ADM | 0.304389965 | 0.003012475 | negative |
| PRIM2 | ADM | 0.627878835 | 1.627E11 | negative |
| PRPS2 | ADM | 0.405577736 | 5.50436E05 | negative |
| PTDSS1 | ADM | 0.400189768 | 7.05197E05 | negative |
| RDH12 | ADM | 0.38713697 | 0.000126315 | negative |
| TRMT11 | ADM | 0.564733796 | 3.71798E09 | negative |
| POLR3F | PPFIA4 | 0.438022268 | 1.1283E05 | negative |
| PRIM2 | PPFIA4 | 0.531042877 | 4.34906E08 | negative |
| PRPS1 | PPFIA4 | 0.309648097 | 0.002524791 | negative |
| PRPS2 | PPFIA4 | 0.497427265 | 3.92702E07 | negative |
| PTDSS1 | PPFIA4 | 0.576138036 | 1.51706E09 | negative |
| SMS | PPFIA4 | 0.439937863 | 1.02221E05 | negative |
| TRMT11 | PPFIA4 | 0.340421066 | 0.000841372 | negative |
| PPOX | TPBG | 0.301727756 | 0.003290276 | negative |
| PTDSS1 | TPBG | 0.306097534 | 0.002845555 | negative |
| SMS | TPBG | 0.512727783 | 1.48532E07 | negative |
| HADH | ADM | 0.43793694 | 1.13326E05 | negative |
| HCCS | ADM | 0.507501437 | 2.08149E07 | negative |
| INPP1 | ADM | 0.383006791 | 0.000151141 | negative |
| LCMT1 | ADM | 0.44100438 | 9.67277E06 | negative |
| MDH1 | ADM | 0.475729502 | 1.44115E06 | negative |
| MGST3 | ADM | 0.425427164 | 2.12831E05 | negative |
| NAGK | ADM | 0.313621856 | 0.002204667 | negative |
| NIT2 | ADM | 0.552876567 | 9.11461E09 | negative |
| GSTO1 | PPFIA4 | 0.446215403 | 7.36498E06 | negative |
| HADH | PPFIA4 | 0.308000971 | 0.002669315 | negative |
| HADHB | PPFIA4 | 0.383766833 | 0.000146258 | negative |
| HCCS | PPFIA4 | 0.487533768 | 7.18366E07 | negative |
| INPP1 | PPFIA4 | 0.503227089 | 2.73166E07 | negative |
| LCMT1 | PPFIA4 | 0.651880491 | 1.47101E12 | negative |
| LDHA | PPFIA4 | 0.49491972 | 4.58481E07 | negative |
| MDH1 | PPFIA4 | 0.668155662 | 2.54106E13 | negative |
| MGST3 | PPFIA4 | 0.438103674 | 1.12359E05 | negative |
| NIT2 | PPFIA4 | 0.445061958 | 7.82604E06 | negative |
| GSTO1 | TPBG | 0.308379025 | 0.002635501 | negative |
| GUK1 | TPBG | 0.363068513 | 0.000347801 | negative |
| HADHB | TPBG | 0.365353702 | 0.000316971 | negative |
| HCCS | TPBG | 0.359069036 | 0.000408474 | negative |
| INPP1 | TPBG | 0.405126687 | 5.62064E05 | negative |
| INPPL1 | TPBG | 0.32228538 | 0.001629999 | negative |
| LCMT1 | TPBG | 0.519403696 | 9.57223E08 | negative |
| LDHA | TPBG | 0.433752495 | 1.4031E05 | negative |
| MARS1 | TPBG | 0.396428231 | 8.3627E05 | negative |
| MDH1 | TPBG | 0.53663657 | 2.94507E08 | negative |
| MGST3 | TPBG | 0.390634339 | 0.000108308 | negative |
| NIT2 | TPBG | 0.332661499 | 0.001122009 | negative |
| NUDT9 | ADM | 0.378422105 | 0.000183942 | negative |
| PAFAH1B2 | ADM | 0.443499881 | 8.49382E06 | negative |
| PAICS | ADM | 0.413220911 | 3.84476E05 | negative |
| PCCB | ADM | 0.492120434 | 5.4422E07 | negative |
| PDHA1 | ADM | 0.468957911 | 2.12392E06 | negative |
| PIP5K1B | ADM | 0.447921885 | 6.72946E06 | negative |
| PLCG2 | ADM | 0.498968278 | 3.56831E07 | negative |
| POLR1C | ADM | 0.32175511 | 0.001660831 | negative |
| POLR1E | ADM | 0.402534489 | 6.33447E05 | negative |
| POLR1H | ADM | 0.43796045 | 1.13189E05 | negative |
| POLR3B | FAM162A | 0.330473513 | 0.001215252 | negative |
| NUDT9 | PPFIA4 | 0.384410079 | 0.00014224 | negative |
| PAFAH1B2 | PPFIA4 | 0.550682955 | 1.07184E08 | negative |
| PAICS | PPFIA4 | 0.458293681 | 3.84762E06 | negative |
| PCCB | PPFIA4 | 0.549348767 | 1.18222E08 | negative |
| PDHA1 | PPFIA4 | 0.467145957 | 2.35287E06 | negative |
| PIK3CG | PPFIA4 | 0.365752387 | 0.000311858 | negative |
| PIKFYVE | PPFIA4 | 0.386093615 | 0.000132202 | negative |
| PIP5K1B | PPFIA4 | 0.477156203 | 1.32667E06 | negative |
| PLCG2 | PPFIA4 | 0.523196737 | 7.42621E08 | negative |
| POLR1C | PPFIA4 | 0.353892828 | 0.000501424 | negative |
| POLR1E | PPFIA4 | 0.470944206 | 1.89717E06 | negative |
| POLR1H | PPFIA4 | 0.413812624 | 3.73806E05 | negative |
| POLR3C | PPFIA4 | 0.374188472 | 0.000219958 | negative |
| NUDT9 | TPBG | 0.301425334 | 0.003323242 | negative |
| OCRL | TPBG | 0.432694914 | 1.48026E05 | negative |
| PAFAH1B2 | TPBG | 0.391552232 | 0.000103993 | negative |
| PAICS | TPBG | 0.410859879 | 4.29945E05 | negative |
| PDHA1 | TPBG | 0.341958389 | 0.000794032 | negative |
| PIK3C3 | TPBG | 0.317185678 | 0.001949195 | negative |
| PIK3CA | TPBG | 0.341532242 | 0.000806905 | negative |
| PIK3CG | TPBG | 0.541453341 | 2.09328E08 | negative |
| PIKFYVE | TPBG | 0.518566885 | 1.01194E07 | negative |
| PIP5K1B | TPBG | 0.402978243 | 6.20657E05 | negative |
| PLA2G4A | TPBG | 0.335264973 | 0.001019558 | negative |
| PLCG2 | TPBG | 0.343057479 | 0.000761691 | negative |
| POLD3 | TPBG | 0.37497002 | 0.000212855 | negative |
| POLR1C | TPBG | 0.506356248 | 2.23955E07 | negative |
| ACP4 | ADM | 0.497339462 | 3.94846E07 | positive |
| ADCY2 | ADM | 0.311720028 | 0.002353007 | positive |
| ADSS1 | ADM | 0.720683014 | 3.81136E16 | positive |
| AK4 | ADM | 0.338419709 | 0.000906857 | positive |
| ALLC | ADM | 0.626380767 | 1.87745E11 | positive |
| AMDHD2 | ADM | 0.600238076 | 2.02923E10 | positive |
| AOC1 | ADM | 0.458210741 | 3.86514E06 | positive |
| AOC2 | ADM | 0.556094162 | 7.17074E09 | positive |
| AOX1 | ADM | 0.394599694 | 9.07856E05 | positive |
| CA4 | ADM | 0.451967794 | 5.42269E06 | positive |
| CKMT1B | ADM | 0.35817904 | 0.000423235 | positive |
| ACO2 | FAM162A | 0.389559035 | 0.000113573 | positive |
| ACP1 | FAM162A | 0.451334094 | 5.61022E06 | positive |
| ADCY7 | FAM162A | 0.450737947 | 5.7922E06 | positive |
| CHST11 | FAM162A | 0.414102091 | 3.68688E05 | positive |
| ACP4 | PPFIA4 | 0.61090105 | 7.89037E11 | positive |
| ADSS1 | PPFIA4 | 0.808440479 | 1.15691E22 | positive |
| AFMID | PPFIA4 | 0.325729243 | 0.001442045 | positive |
| AK4 | PPFIA4 | 0.454146756 | 4.82191E06 | positive |
| ALLC | PPFIA4 | 0.844089278 | 2.30096E26 | positive |
| AMDHD2 | PPFIA4 | 0.680240097 | 6.41485E14 | positive |
| AOC1 | PPFIA4 | 0.350871867 | 0.000564261 | positive |
| AOC2 | PPFIA4 | 0.690790228 | 1.82603E14 | positive |
| AOX1 | PPFIA4 | 0.389954216 | 0.000111611 | positive |
| BCO1 | PPFIA4 | 0.425833163 | 2.08603E05 | positive |
| CA4 | PPFIA4 | 0.591988967 | 4.11611E10 | positive |
| CKMT1B | PPFIA4 | 0.561993797 | 4.58837E09 | positive |
| ACP4 | TPBG | 0.401363344 | 6.6839E05 | positive |
| ADSS1 | TPBG | 0.637061938 | 6.64959E12 | positive |
| ALLC | TPBG | 0.69738069 | 8.10414E15 | positive |
| AMDHD2 | TPBG | 0.591223002 | 4.39106E10 | positive |
| AOC2 | TPBG | 0.685764153 | 3.34448E14 | positive |
| AOX1 | TPBG | 0.39653783 | 8.3215E05 | positive |
| BCO1 | TPBG | 0.360789753 | 0.000381269 | positive |
| CA4 | TPBG | 0.511991138 | 1.5582E07 | positive |
| CKMT1B | TPBG | 0.546032972 | 1.50551E08 | positive |
| CMAS | ADM | 0.431204175 | 1.59581E05 | positive |
| CTH | ADM | 0.568664105 | 2.74037E09 | positive |
| CYP2E1 | ADM | 0.529658007 | 4.78453E08 | positive |
| DGAT2 | ADM | 0.345058615 | 0.000705878 | positive |
| DHDH | ADM | 0.375623318 | 0.00020708 | positive |
| ENPP1 | ADM | 0.474618749 | 1.53669E06 | positive |
| ENPP7 | ADM | 0.496779561 | 4.08778E07 | positive |
| FMO1 | ADM | 0.438859077 | 1.08075E05 | positive |
| FTMT | ADM | 0.544174425 | 1.722E08 | positive |
| GDA | ADM | 0.388012153 | 0.000121566 | positive |
| GLYCTK | ADM | 0.54830139 | 1.27639E08 | positive |
| GNMT | ADM | 0.401302404 | 6.70256E05 | positive |
| GPT | ADM | 0.575656437 | 1.57669E09 | positive |
| DGKD | FAM162A | 0.322417216 | 0.001622414 | positive |
| DGKE | FAM162A | 0.35988349 | 0.000395382 | positive |
| GPD1L | FAM162A | 0.326696727 | 0.001392901 | positive |
| CMAS | PPFIA4 | 0.548038586 | 1.30112E08 | positive |
| CPT1C | PPFIA4 | 0.302330449 | 0.00322545 | positive |
| CTH | PPFIA4 | 0.697600277 | 7.88476E15 | positive |
| CYP2A13 | PPFIA4 | 0.383543278 | 0.000147679 | positive |
| CYP2E1 | PPFIA4 | 0.546572448 | 1.4477E08 | positive |
| DGAT2 | PPFIA4 | 0.302253902 | 0.003233619 | positive |
| DHDH | PPFIA4 | 0.492186413 | 5.42035E07 | positive |
| ENPP1 | PPFIA4 | 0.634918891 | 8.21556E12 | positive |
| ENPP7 | PPFIA4 | 0.583451298 | 8.38302E10 | positive |
| FMO1 | PPFIA4 | 0.403038097 | 6.1895E05 | positive |
| FTMT | PPFIA4 | 0.559219512 | 5.66647E09 | positive |
| GDA | PPFIA4 | 0.593340517 | 3.67075E10 | positive |
| GLYCTK | PPFIA4 | 0.475516169 | 1.45906E06 | positive |
| GNMT | PPFIA4 | 0.310175589 | 0.002480019 | positive |
| GPD1 | PPFIA4 | 0.342557466 | 0.000776252 | positive |
| GPT | PPFIA4 | 0.68032481 | 6.35177E14 | positive |
| CMAS | TPBG | 0.537483277 | 2.7746E08 | positive |
| CTH | TPBG | 0.649309265 | 1.92258E12 | positive |
| CYP2A13 | TPBG | 0.406863263 | 5.18513E05 | positive |
| CYP2E1 | TPBG | 0.483271643 | 9.26456E07 | positive |
| DGAT2 | TPBG | 0.322786011 | 0.001601366 | positive |
| DGKA | TPBG | 0.314508734 | 0.002138418 | positive |
| DHDH | TPBG | 0.309931475 | 0.002500649 | positive |
| ENPP1 | TPBG | 0.632481214 | 1.0429E11 | positive |
| ENPP7 | TPBG | 0.45441873 | 4.75148E06 | positive |
| FTMT | TPBG | 0.581978196 | 9.45805E10 | positive |
| GDA | TPBG | 0.584217248 | 7.87138E10 | positive |
| GPT | TPBG | 0.517394143 | 1.09365E07 | positive |
| PTEN | ADM | 0.334676352 | 0.001041946 | positive |
| PTGDS | ADM | 0.410428101 | 4.38784E05 | positive |
| QPRT | ADM | 0.304534835 | 0.00299798 | positive |
| RDH8 | ADM | 0.597384776 | 2.59752E10 | positive |
| SPHK1 | ADM | 0.415290115 | 3.48359E05 | positive |
| SUOX | ADM | 0.391717313 | 0.000103234 | positive |
| SYNJ2 | ADM | 0.356609654 | 0.000450462 | positive |
| TBXAS1 | ADM | 0.448614293 | 6.48661E06 | positive |
| TPO | ADM | 0.542278678 | 1.97327E08 | positive |
| UPB1 | ADM | 0.669006083 | 2.31129E13 | positive |
| UPP1 | ADM | 0.490768457 | 5.90877E07 | positive |
| PRPS1 | FAM162A | 0.309091664 | 0.002572807 | positive |
| SHMT2 | FAM162A | 0.520734738 | 8.7595E08 | positive |
| SPHK1 | FAM162A | 0.355691124 | 0.000467134 | positive |
| UAP1 | FAM162A | 0.324749303 | 0.001493419 | positive |
| PTGDS | PPFIA4 | 0.418052629 | 3.05063E05 | positive |
| RDH8 | PPFIA4 | 0.417186629 | 3.18062E05 | positive |
| SULT2B1 | PPFIA4 | 0.35847171 | 0.000418328 | positive |
| SUOX | PPFIA4 | 0.479839506 | 1.13426E06 | positive |
| SYNJ2 | PPFIA4 | 0.482015205 | 9.97952E07 | positive |
| TBXAS1 | PPFIA4 | 0.486371282 | 7.70241E07 | positive |
| TPO | PPFIA4 | 0.677340667 | 8.97846E14 | positive |
| UPB1 | PPFIA4 | 0.55484922 | 7.87048E09 | positive |
| SULT2B1 | TPBG | 0.421021251 | 2.64172E05 | positive |
| SUOX | TPBG | 0.361746037 | 0.000366879 | positive |
| SYNJ2 | TPBG | 0.362910595 | 0.00035003 | positive |
| TBXAS1 | TPBG | 0.446936761 | 7.08974E06 | positive |
| TPO | TPBG | 0.585981621 | 6.80425E10 | positive |
| UPB1 | TPBG | 0.477040157 | 1.33565E06 | positive |
| GPX5 | ADM | 0.344722832 | 0.000714974 | positive |
| GSTM5 | ADM | 0.499404619 | 3.47253E07 | positive |
| GSTZ1 | ADM | 0.377722283 | 0.000189492 | positive |
| ITPKA | ADM | 0.318428555 | 0.001866591 | positive |
| LPL | ADM | 0.317499733 | 0.001928016 | positive |
| LYPLA2 | ADM | 0.367118504 | 0.000294906 | positive |
| NNMT | ADM | 0.538417269 | 2.59748E08 | positive |
| NPR2 | ADM | 0.446593477 | 7.21949E06 | positive |
| GPX4 | FAM162A | 0.367735312 | 0.000287534 | positive |
| GSTK1 | FAM162A | 0.408426541 | 4.82018E05 | positive |
| GSTP1 | FAM162A | 0.456464429 | 4.25197E06 | positive |
| GUK1 | FAM162A | 0.517516393 | 1.08485E07 | positive |
| MARS1 | FAM162A | 0.48494583 | 8.38699E07 | positive |
| GPX5 | PPFIA4 | 0.301053937 | 0.003364132 | positive |
| GSTM5 | PPFIA4 | 0.681309264 | 5.66137E14 | positive |
| INPP5J | PPFIA4 | 0.355401888 | 0.0004725 | positive |
| ITPKA | PPFIA4 | 0.495681301 | 4.37472E07 | positive |
| LYPLA2 | PPFIA4 | 0.43384925 | 1.39623E05 | positive |
| NAGS | PPFIA4 | 0.411394496 | 4.1923E05 | positive |
| NNMT | PPFIA4 | 0.612568708 | 6.78497E11 | positive |
| NPR1 | PPFIA4 | 0.446557094 | 7.23337E06 | positive |
| NPR2 | PPFIA4 | 0.64040299 | 4.76622E12 | positive |
| GSTM5 | TPBG | 0.655316346 | 1.02447E12 | positive |
| INPP5J | TPBG | 0.379409635 | 0.000176366 | positive |
| ITPKA | TPBG | 0.391686187 | 0.000103377 | positive |
| NNMT | TPBG | 0.437069843 | 1.18482E05 | positive |
| NPR1 | TPBG | 0.479098173 | 1.1846E06 | positive |
| NPR2 | TPBG | 0.694293685 | 1.18887E14 | positive |
| OTC | ADM | 0.516882832 | 1.13121E07 | positive |
| PDE1B | ADM | 0.340056536 | 0.000852968 | positive |
| PDE6C | ADM | 0.636887703 | 6.76532E12 | positive |
| PDHA2 | ADM | 0.452394299 | 5.29981E06 | positive |
| PGM1 | ADM | 0.548280593 | 1.27833E08 | positive |
| PGS1 | ADM | 0.428650338 | 1.81362E05 | positive |
| PLA2G15 | ADM | 0.361546471 | 0.00036984 | positive |
| PLA2G5 | ADM | 0.420887656 | 2.65896E05 | positive |
| PLCB3 | ADM | 0.422707822 | 2.43285E05 | positive |
| PLCD4 | ADM | 0.487199159 | 7.32948E07 | positive |
| PNLIPRP1 | ADM | 0.502942862 | 2.78112E07 | positive |
| POLR2F | ADM | 0.331323289 | 0.001178233 | positive |
| PAICS | FAM162A | 0.34332616 | 0.00075397 | positive |
| PDE1B | FAM162A | 0.37489569 | 0.000213521 | positive |
| PDE4B | FAM162A | 0.35886231 | 0.00041186 | positive |
| PIK3C3 | FAM162A | 0.329477284 | 0.001259995 | positive |
| PIK3CA | FAM162A | 0.499458835 | 3.46081E07 | positive |
| PIK3CG | FAM162A | 0.5115619 | 1.60223E07 | positive |
| PIKFYVE | FAM162A | 0.307062509 | 0.002754943 | positive |
| PLA2G15 | FAM162A | 0.367068055 | 0.000295517 | positive |
| OTC | PPFIA4 | 0.684685622 | 3.80227E14 | positive |
| PDE6B | PPFIA4 | 0.386440503 | 0.000130217 | positive |
| PDE6C | PPFIA4 | 0.781522601 | 2.3953E20 | positive |
| PDHA2 | PPFIA4 | 0.671541282 | 1.73918E13 | positive |
| PGM1 | PPFIA4 | 0.457130099 | 4.1004E06 | positive |
| PLA2G5 | PPFIA4 | 0.398822176 | 7.50465E05 | positive |
| PLCB3 | PPFIA4 | 0.388247877 | 0.000120315 | positive |
| PLCD4 | PPFIA4 | 0.779913597 | 3.21635E20 | positive |
| PNLIPRP1 | PPFIA4 | 0.713624179 | 9.93409E16 | positive |
| POLA1 | PPFIA4 | 0.300533218 | 0.003422221 | positive |
| POLR2F | PPFIA4 | 0.487614305 | 7.14897E07 | positive |
| OTC | TPBG | 0.623108249 | 2.56008E11 | positive |
| PDE6B | TPBG | 0.341091411 | 0.000820422 | positive |
| PDE6C | TPBG | 0.639386171 | 5.27685E12 | positive |
| PDE6H | TPBG | 0.411484105 | 4.17459E05 | positive |
| PDHA2 | TPBG | 0.628595986 | 1.5188E11 | positive |
| PLA2G5 | TPBG | 0.442263636 | 9.05988E06 | positive |
| PLCD4 | TPBG | 0.640003679 | 4.9608E12 | positive |
| PNLIPRP1 | TPBG | 0.745668373 | 1.00001E17 | positive |

## Supplementary Table 2

**Supplementary Table 2.** Primer sequences used in this paper

|  | Species | Forward | Reverse |
| --- | --- | --- | --- |
| GAPDH | Human | 5’GGAAGCTTGTCATCAATGGAAATC’ | 5’TGATGACCCTTTTGGCTCCC3’ |
| ADM | Human | 5’TTGTCCTCCCCTATTTTAAGACG3’ | 5’CTTCCACACAGGAGGTAATCAGTC3’ |
| PPFIA4 | Human | 5'CCTAACAACAACCGTGACTGAACT3' | 5'CCAGTGTAGTAATCCGCTCTTCC3' |
| TPBG | Human | 5’TGAGCCTGACCTACGTGTC3’ | 5’GCCATTGTGAAGGACCTTGAG3’ |
| FAM162A | Human | 5’ACTGTCTCGTTGGAGATGCTTGATG3’ | 5’CTACCACCGTCAGGGCAATCATTAG3’ |
| GAPDH | Mouse | 5'CCTCGTCCCGTAGACAAAATG3' | 5'TGAGGTCAATGAAGGGGTCGT3' |
| ADM | Mouse | 5’GTGCTGACGGGA TCGTGCTG3’ | 5’CATGCAGTACCCGAGGGACCT3’ |
| PPFIA4 | Mouse | 5’CCCCTGACCCGACGCTGAGA3’ | 5’CGGGCTTGAGGACGGGTAGTTG3’ |
| TPBG | Mouse | 5'GACAGAGGTGGTGCCAGATAAAGC3' | 5'AGCGTCACAGTCCAGGTCAGAG3' |
| FAM162A | Mouse | 5'CTGGCGGCTGGACATTGTCTTAG3' | 5'CCTGTGGCTTGGTGCAGAATCC3' |
